# The Ageing Brain: Effects on DNA Repair and DNA Methylation in Mice

**DOI:** 10.3390/genes8020075

**Published:** 2017-02-17

**Authors:** Sabine A. S. Langie, Kerry M. Cameron, Gabriella Ficz, David Oxley, Bartłomiej Tomaszewski, Joanna P. Gorniak, Lou M. Maas, Roger W. L. Godschalk, Frederik J. van Schooten, Wolf Reik, Thomas von Zglinicki, John C. Mathers

**Affiliations:** 1 Centre for Ageing and Vitality, Human Nutrition Research Centre, Institute of Cellular Medicine, Newcastle University, Campus for Ageing and Vitality, Newcastle upon Tyne NE4 5PL, UK; tomaszewski.bartlomiej@gmail.com (B.T.); joanna.p.gorniak@googlemail.com (J.P.G.); john.mathers@ncl.ac.uk (J.C.M.); 2 The Ageing Biology Centre and Institute for Cell and Molecular Biology, Newcastle University, Campus for Ageing and Vitality, Newcastle upon Tyne NE4 5PL, UK; kerry.m.cameron@effem.com (K.M.C.); t.vonzglinicki@newcastle.ac.uk (T.v.Z.); 3 Barts Cancer Institute, Queen Mary University, London EC1M 6BQ, UK; g.ficz@qmul.ac.uk; 4 Mass Spectrometry Laboratory, Babraham Institute, Cambridge CB22 3AT, UK; david.oxley@babraham.ac.uk; 5 Department of Pharmacology & Toxicology, School for Nutrition and Translational Research in Metabolism (NUTRIM), Maastricht University, 6200 MD Maastricht, The Netherlands; l.maas@maastrichtuniversity.nl (L.M.M.); r.godschalk@maastrichtuniversity.nl (R.W.L.G.); f.vanschooten@maastrichtuniversity.nl (F.J.v.S.); 6 Epigenetics Programme, Babraham Institute, Cambridge CB22 3AT, UK; wolf.reik@babraham.ac.uk; 7 Wellcome Trust Sanger Institute, Hinxton CB10 1SA, UK

**Keywords:** DNA methylation, epigenetics, base excision repair, ageing, brain, gene regulation

## Abstract

Base excision repair (BER) may become less effective with ageing resulting in accumulation of DNA lesions, genome instability and altered gene expression that contribute to age-related degenerative diseases. The brain is particularly vulnerable to the accumulation of DNA lesions; hence, proper functioning of DNA repair mechanisms is important for neuronal survival. Although the mechanism of age-related decline in DNA repair capacity is unknown, growing evidence suggests that epigenetic events (e.g., DNA methylation) contribute to the ageing process and may be functionally important through the regulation of the expression of DNA repair genes. We hypothesize that epigenetic mechanisms are involved in mediating the age-related decline in BER in the brain. Brains from male mice were isolated at 3–32 months of age. Pyrosequencing analyses revealed significantly increased *Ogg1* methylation with ageing, which correlated inversely with *Ogg1* expression. The reduced *Ogg1* expression correlated with enhanced expression of methyl-CpG binding protein 2 and ten-eleven translocation enzyme 2. A significant inverse correlation between *Neil1* methylation at CpG-site2 and expression was also observed. BER activity was significantly reduced and associated with increased 8-oxo-7,8-dihydro-2′-deoxyguanosine levels. These data indicate that *Ogg1* and *Neil1* expression can be epigenetically regulated, which may mediate the effects of ageing on DNA repair in the brain.

## 1. Introduction

Ageing is associated with the accumulation of oxidative DNA damage resulting from increased exposure to reactive oxygen species (ROS) from exogenous and endogenous sources [1]. DNA is subject to constant attack by DNA-damaging agents, and the accumulation of unrepaired DNA damage has profound effects on cell function. This may cause the characteristic features of ageing, including changes in gene expression, genome instability, changes in cell replication, cell senescence and cell death [2]. The DNA base guanine is particularly sensitive to oxidation by ROS to form 8-oxo-7,8-dihydro-2′-deoxyguanosine (8-oxodG) due to its low redox potential (reviewed by [3]). The DNA glycosylase oxoguanosine 1 (OGG1) is the major base excision repair (BER) enzyme, which recognizes and removes 8-oxodG from DNA. Other DNA glycosylases of interest are: (i) Nei endonuclease VIII-like 1 (NEIL1), which recognizes and incises 8-oxodG lesions located near the 3′-end of single strand breaks, DNA bubble structures and single-stranded structures where OGG1 has limited activity [4]; and (ii) muty homolog (MUTYH), which has a specificity for adenine opposite 8-oxoguanine [5]. The DNA ligase and X-ray repair cross-complementing protein 1 (XRCC1) complex complete the repair process by sealing the nick. Interestingly, OGG1 interacts with XRCC1, enhancing its incision activity [6]. Whilst most oxidative damage is removed by BER and other DNA repair mechanisms, it is thought that these mechanisms become less effective with ageing, resulting in the accumulation of DNA lesions, loss of genome stability and altered gene expression that contribute to age-related degenerative diseases. The role of DNA glycosylases dysfunction in ageing may be important, as shown by age-related accumulation of oxidative damage in liver from *Ogg1* KO mice [7]. Furthermore, the activity of human OGG1 declines with age in lymphocytes [8] and is lower in Alzheimer’s disease brains [9,10]. *Neil1* is highly expressed in the brain [11]. Binding between the DNA damage sensor protein poly(ADP-ribose) polymerase 1 (PARP-1) and NEIL1 was diminished in older mice compared with younger mice, supporting the idea of impaired DNA repair during aging [12].

The mechanisms responsible for age-related decline in DNA repair capacity are uncertain, but growing evidence suggests that epigenetic events, including aberrant DNA methylation, contribute to the ageing process and may be functionally important through dysregulation of gene expression of, e.g., DNA repair genes [13,14,15,16,17]. Epigenetics defines processes and genomic markers, including DNA methylation, covalent histone modifications and non-coding RNAs, that result in changes in gene expression and phenotype without a corresponding alteration in DNA sequence, thus providing a process for genome regulation. DNA methylation is the most widely-studied epigenetic mechanism and is achieved by the addition of a methyl group to a cytosine (5mC) in CpG dinucleotides by DNA methyltransferases [18]. CpGs are often densely packed in or close to promoter regions, forming so-called “CpG islands”, which are normally unmethylated in expressed genes [19]. Whilst some epigenetic markers are established during embryonic and foetal development and remain relatively stable during adulthood, the methylation status of some genomic loci is labile and changes over time and in response to environmental exposures [20,21]. Aberrant DNA methylation of CpG sites can inhibit the opportunity for transcription factors (TF) to bind, which can lead to gene silencing [18]. In addition, ROS can cause oxidation of 5mC to 5-hydroxymethylcytosine (5hmC) [22], and although the specific biological role of 5hmC is unclear, it may counteract transcriptional repression, making 5hmC important for gene regulation (reviewed by [23]). Alternatively, ten-eleven translocation (TET) enzymes can convert 5mC to 5hmC and play a role in active DNA demethylation [22,23,24].

Evidence has been demonstrated for age-related changes in DNA methylation in studies of young and older monozygotic twins [25]. Despite the identical genotypes, in older twins, there was greater inter-twin variability in the epigenomes compared with younger twins, and this was accompanied by greater inter-twin diversity in gene expression portraits. Interestingly, global DNA demethylation has been accompanied by hypermethylation of specific gene promoters. Few studies have reported investigations of promoter-specific methylation of genes involved in DNA repair [13,14,15,17,26,27,28]; these have been mainly in relation to cancer, and none have studied epigenetic regulation of BER-related genes in the ageing brain.

In the present study, the brain was selected as the target tissue since it is particularly vulnerable to the deleterious effects of ROS due to its high oxygen utilization and relatively low antioxidants levels [29,30]. DNA damage may be especially harmful in post-mitotic neuronal brain cells, which have limited capacity to regenerate. Thus, oxidative DNA damage may play a key role in age-associated loss of brain neurons; hence, cumulative unrepaired DNA damage may be responsible for the underlying cellular dysfunction [31]. For these reasons, the proper functioning of DNA repair mechanisms is important for neuronal survival.

**Figure 1:**
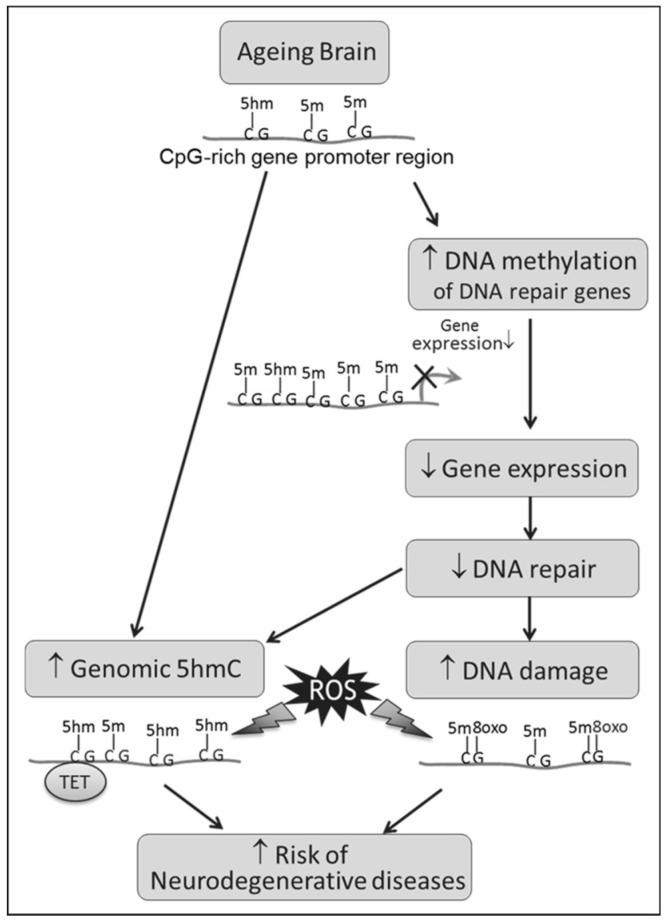
Schematic overview of the study hypothesis. Abbreviations used: 5mC, 5-methylcytosine; 5hmC, 5-hydroxymethycytosine; 8-oxodG, 8-oxo-7,8-dihydro-2′-deoxyguanosine; ROS: reactive oxygen species; TET: ten-eleven translocation enzymes.

We hypothesize that epigenetic mechanisms are involved in mediating the age-related decline in DNA repair in the brain (Figure 1). Thus, through altered gene expression, changes in the methylation status of promoters of genes encoding components of DNA repair systems may impact on neuronal DNA repair. This may lead to the accumulation of oxidative DNA damage and mutations across the whole genome, causing genome instability and increasing the risk of age-related degenerative neurological diseases. To test this hypothesis, we studied: (i) the formation of oxidative DNA damage and global DNA methylation levels; (ii) BER gene expression and the methylation status of CpGs in TF binding sites that influence the transcription of BER genes; and (iii) correlations with the resulting phenotypic BER-related incision activity in male mice across most of the adult lifespan (3–32 months old).

## 2. Materials and Methods

### 2.1. Animals and Design of the Study

Mice were obtained from a long-established colony of the C57/BL (ICRFa) strain, which had been selected for use in studies of intrinsic ageing because it is free from specific age-associated pathologies and, thus, provides a good general model of ageing [32]. Mice were housed in standard cages of different sizes depending on housing density (between 1 and 6 mice per cage). Mice were housed at 20 ± 2 °C under a 12-h light/12-h dark photoperiod with lights on at 7 a.m. Mice were provided with sawdust and paper bedding and had ad libitum access to water and food (CRM (P) Special Diet Services; BP Nutrition Ltd., Essex, U.K.). All work complied with the U.K. Home Office Animals (Scientific procedures) Act of 1986 (project licence PPL60/3864).

To study the effects of ageing, whole brains were collected from ad libitum fed male mice at ages 3, 6, 12, 24, 28, 31 and 32 months (*n* = 4 per age group, except 31 and 32 months, *n* = 3 per group), immediately snap frozen in liquid nitrogen and stored at −80 °C. When required, frozen brain tissues were ground and aliquoted and stored at −80 °C until further analysis.

### 2.2. Determination of 8-oxodG

Frozen ground brain tissues (~30–80 mg, *n* = 3–4 per group) were thawed, and genomic DNA was isolated using standard phenol extraction [33]. The DNA extraction procedure was optimized to minimize artificial induction of 8-oxodG, by using radical-free phenol, minimizing exposure to oxygen and by the addition of 1 mM deferoxamine mesylate and 20 mM TEMPO (2,2,6,6-tetramethylpiperidine-*N*-oxyl; Aldrich, Steinheim, Germany), according to the recommendations made by the European Standards Committee on Oxidative DNA Damage (ESCODD [34]). To detect the base oxidation product 8-oxodG, HPLC with electrochemical detection (ECD) was performed as described earlier [35].

### 2.3. Assessment of Genomic 5mC and 5hmC

Nucleosides were derived from DNA samples (*n* = 3–4 per group) by digestion with DNA Degradase Plus (Zymo Research, Cambridge Bioscience, Cambridge, U.K.) according to the manufacturer’s instructions and were analysed by LC-MS/MS on an LTQ Orbitrap Velos mass spectrometer (Thermo Scientific, Cramlington, UK) fitted with a nanoelectrospray ion-source (Proxeon/Thermo Scientific; Amsterdam, The Netherlands). Mass spectral data for 5hmC, 5mC and C were acquired in selected reaction monitoring (SRM) mode, monitoring the transitions 258 → 142.0611 (5hmC), 242 → 126.0662 (5mC) and 228 → 112.0505 (C). Parent ions were selected for SRM with a 4 mass unit isolation window and fragmented by Higher-energy Collisional Dissociation (HCD) with a relative collision energy of 20%, with R > 14,000 for the fragment ions. Peak areas from extracted ion chromatograms of the relevant fragment ions were quantified by external calibration relative to authentic standards.

### 2.4. Gene-Specific Methylation Studies, Using Pyrosequencing of Bisulphite Converted DNA

Selection of transcription factor (TF) binding sites and Primer design: Genomatix’s “Gene2Promoter” tool (Genomatix; Munich, Germany) was used to retrieve the target genes’ (i.e., *Ogg1*, *Neil1*, *Mutyh* and *Xrcc1*) promoter sequence. Using the free, downloadable CpG Island Explorer 2.0 software ([36]; http://www.soft82.com/download/windows/cpg-island-explorer/), CpG-rich regions were identified in the gene promoters. Next, the CpG-island was screened for TF binding sites by means of the Genomatix “MatInspector” tool. The following selection criteria were applied: core similarity >0.75 and matrix similarity >0.70 (with 1 being a perfect match), and optimized matrix threshold >0.7 (to minimize the number of false positive matches). As advised by Genomatix, both (+)- and (−)-strand matches have been considered equally, since most TF binding sites can occur in both orientations in promoters or enhancers. Moreover, methylation patterns on the (+)- and (−)-strand are believed to be identical since hemi-methylated DNA is restored to the fully-methylated state during DNA replication [37]. All TF binding sites were subsequently filtered based on the association of their TF family with specific tissues (based on MatInspector output); selecting those who are ubiquitously expressed or specifically expressed in the brain/central nervous system/neurons. As a second screening, only those TF that have at least one CpG di-nucleotide in their binding sequence and preferably in their core sequence (i.e., the highest conserved, consecutive positions of the TF) were selected. Based on these screening steps, CpG-sites located in TF binding sites with potential to influence promoter function were selected (Supplementary Materials Figures S1–S4). Using the PSQ Software program (Qiagen, Manchester, U.K.), primers were designed for several amplicons to include all selected CpG sites (Table 1 and Supplementary Materials Figures S1–S4).

Bisulphite conversion: DNA was extracted and purified (including RNase treatment) from ~20 mg of ground tissue using standard chloroform:isoamyl alcohol extraction. Bisulphite conversion of DNA was performed using the EZ DNA Methylation Gold™ kit (Zymo Research, Cambridge Bioscience, Cambridge, U.K.) according to the manufacturer’s protocol.

Pyrosequencing: Bisulphite pyrosequencing was used to quantify methylation at individual CpG sites within the specific TF binding sites. About 50 ng of bisulphite-treated DNA were added as a template in PCR reaction containing 12.5 µL Hot Start Taq Master Mix (Qiagen, Manchester, U.K.), 400 nM forward primer and 400 nM biotin-labelled reverse primer in a total volume of 25 µL. The primer sequences and PCR conditions are summarized in Table 1. Amplification was carried out in a Bio-Rad thermocycler (Bio-Rad, Hertfordshire, U.K.) using the following protocol; 95 °C 15 min, then 50 cycles of 95 °C 15 s, annealing temperature for 30 s (Table 1), 72 °C for 30 s, followed by 72 °C for 5 min. Next, the biotin-labelled PCR products were captured with Streptavidin Sepharose beads (GE Healthcare, Amersham U.K.) and made single stranded using a Pyrosequencing Vacuum Prep Tool (Qiagen). Sequencing primer (Table 1) was annealed to the single-stranded PCR product by heating to 80 °C, followed by slow cooling. Pyrosequencing was then carried out on a Pyromark MD system (Qiagen). Each sample was run in duplicate, and cytosine methylation was quantified by the Pyro Q CpG 1.0.6 software (Qiagen, Manchester, U.K.). If poor quality data were obtained for both duplicates or the assay failed (flagged in red by the software), that sample was omitted from further data analysis, which was the case for 3 samples when running the *Ogg1* pyrosequencing analyses and 4 samples in the case of *Neil1*.

### 2.5. Gene Expression Analyses

Total RNA was extracted from brain samples (~20 mg) using TRIzol reagent (Ambion, Life Technologies, Paisley, U.K.) according to the manufacturer’s protocol. Next, 500 ng of total DNase-treated RNA were used for reverse transcription with the RevertAid™ H Minus First Strand cDNA Synthesis Kit (Fermentas, Thermo Scientific, Cramlington, U.K.) at 45 °C for 1 h.

Real-time quantitative reverse transcription (RT)-PCR of cDNAs derived from specific transcripts was performed in a Light Cycler 480 (Roche Diagnostics, Mannheim, Germany) using the respective pairs of oligonucleotide primers (Table 2). cDNA (~25 ng) was mixed with 12.5 µL Maxima™ SYBR Green qPCR Master Mix (Fermentas), 0.75 pmol of the forward and 0.75 pmol of the reverse primer of our genes of interest, and RNase-free water was added to achieve an end volume of 25 µL. Amplification was carried out using the following protocol; 95 °C for 10 min, then 45 cycles of 95 °C for 15 s and annealing temperature 60 °C for 1 min, with signal acquisition at extension steps. To confirm amplification specificity, the PCR products from each primer pair were subjected to melt curve analysis and agarose gel electrophoresis. Each sample was analysed in triplicate, and LightCycler 480 software release 1.5.0 (Roche Diagnostics, Mannheim, Germany) was used for data analysis. Expression of *Ogg1*, *Neil1*, *Mutyh* and *Xrcc1* was normalized relative to that of control transcripts *HPRT* and *β-microglobulin*, while the expression of *Tet1–3* and *Mecp2* was normalized to *Atp5b* and *Gapdh*. Levels of expressions, also called Relative quantification (RQ) values, were obtained by the 2^−∆∆Ct^ method. These RQ values were subsequently log2 transformed to give the symmetric fold change.

### 2.6. Measurement of BER-Related DNA Incision Activity

BER activity in brain tissues was assessed using a modified comet-based repair assay that was recently optimized for the use of tissue extracts [38]. This assay measures the ability of BER-related enzymes that are present in tissue extracts to recognize and incise substrate DNA containing 8-oxodG lesions that were induced by the photosensitizer Ro 19-8022 plus light.

The protocol has been described in full detail before [38]. Briefly, to prepare tissue extracts, ~30-mg aliquots of ground tissue were incubated with 75 µL Buffer A (45 mM HEPES, 0.4 M KCl, 1 mM EDTA, 0.1 mM dithiothreitol, 10% glycerol, adjusted to pH 7.8 (all purchased from Sigma, Dorset, U.K.)), vortexed vigorously, snap frozen in liquid nitrogen and immediately defrosted. Next, 30 µL of 1% Triton X-100 in Buffer A (Sigma, Dorset, U.K.) was added per 100-µL aliquot and incubated on ice for 10 min. After, centrifugation at 14,000× *g* for 5 min at 4 °C to remove cell debris, the supernatant was collected, and protein concentrations were determined by the Bio-Rad DC Protein Assay Kit using bovine serum albumin as a standard and controlling for the presence of Triton X-100. Final protein extracts were diluted with 0.23% Triton X-100 in Buffer A to a concentration of 1 mg/mL before further use in the repair incubation. Comets were visualized using an Olympus BX51 fluorescence microscope, and 50 comets/slide selected at random were analysed using the Comet assay IV software program (Perceptive Instruments, Haverhill, U.K.). %DNA in the tail (also known as tail intensity (TI)) was used for further calculations. After subtracting background levels from all data, the final repair capacity was calculated according to Langie et al. [38].

### 2.7. Statistical Analysis

Results are presented as the mean values ± standard error. Grubbs’ test, also called the ESD method (extreme Studentized deviate), was used to determine significant outliers within the 8-oxodG dataset; data from 5 samples were omitted. Differences in levels of genomic 5mC and 5hmC, gene-specific DNA methylation, gene expression, BER-related incision activity and oxidative DNA damage were analysed by ANOVA, using Dunnett’s *t*-test when comparing treatments. Relationships between variables were assessed by regression analyses, conducting multiple linear stepwise regression analysis when studying the effect of the various individual CpG-sites in TF binding sites on gene expression. Statistical analysis was performed using SPSS v.19.0 (IBM), and *p* < 0.05 was considered statistically significant.

## 3. Results

Our findings are structured according to the steps indicated in the schematic overview of our study hypothesis (Figure 1).

### 3.1. The Effect of Ageing on Genome Stability, DNA Damage and DNA Methylation

**Figure 2:**
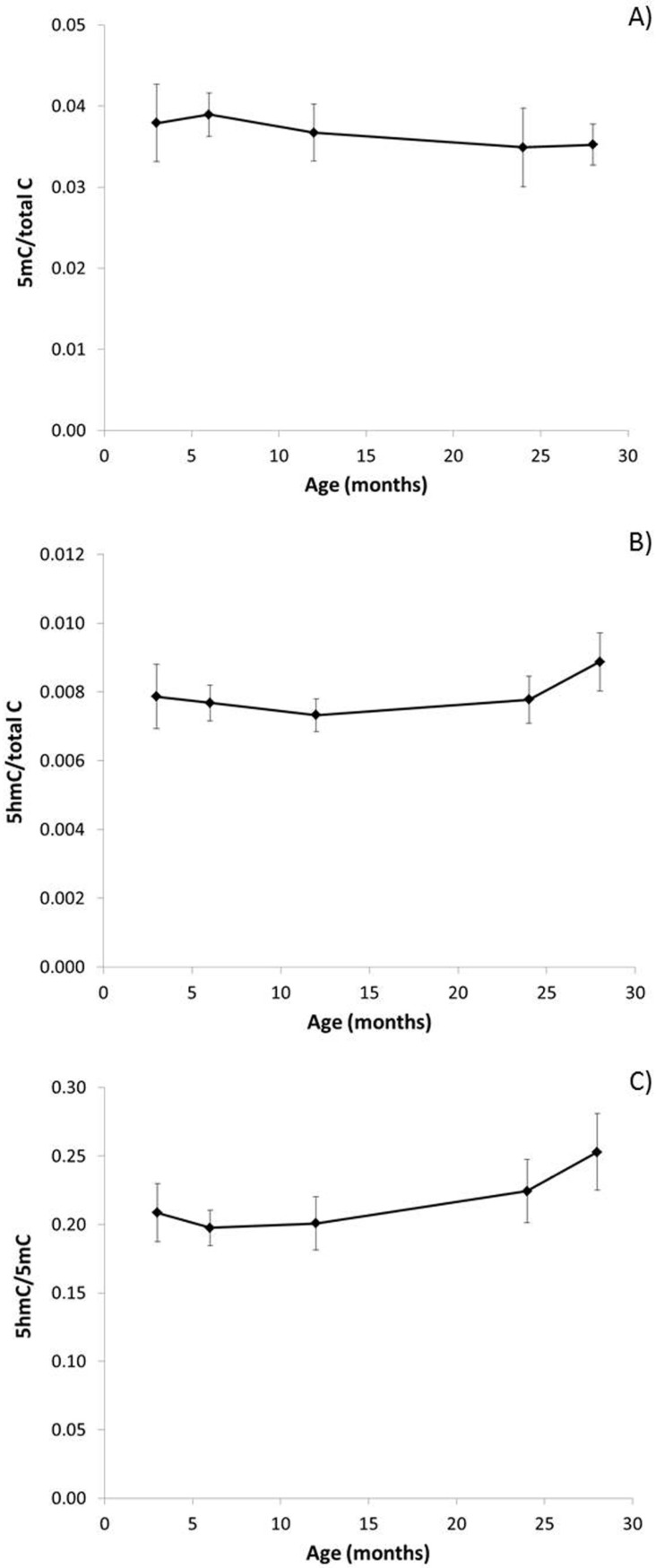
Effect of ageing on genomic 5mC and 5hmC levels. Levels of: (**A**) genomic 5mC over total cytosine; (**B**) levels of 5hmC over total cytosine; and (**C**) ratio of 5hmC/mC (*P*_ANOVA_ = 0.027; R^2^ = 0.382, *p* = 0.008) in the brain of male mice. Percentages shown are calculated from the mean values (*n* = 4), and error bars represent standard errors of the mean (SEM).

**Figure 3:**
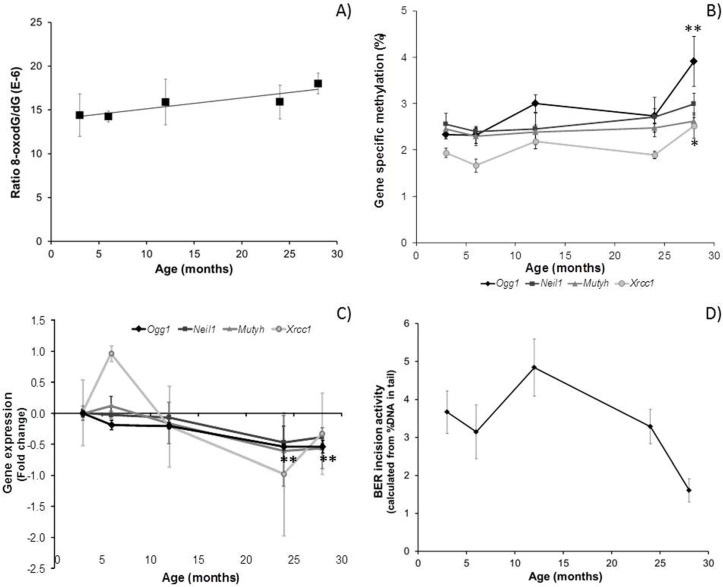
Effect of ageing on: (**A**) levels of 8-oxodG (R^2^ = 0.854, *p* = 0.025); (**B**) BER-related gene promotor methylation (*Ogg1*: *P*_ANOVA_ = 0.026, ** *P*_(28 vs. 3 months)_ = 0.015; *Xrcc1*: *P*_ANOVA_ = 0.023, * *P*_(28 vs. 3 months)_ = 0.041); (**C**) BER-related gene expression (*Ogg1*: *P*_ANOVA_ < 0.001, ** *P*_(24 vs. 3 months)_ < 0.001, ** *P*_(28 vs. 3 months)_ < 0.001); and (**D**) BER-related incision activity (*P*_ANOVA_ = 0.021) in mouse brain. Data are presented as the mean values (*n* = 3–4), and bars indicate SEM.

Ageing was associated with decreased global DNA methylation (5mC) (Figure 2A) and increased 5hmC (Figure 2B), though these trends were not statistically significant. However, there was a significant effect of age on the 5hmC/5mC ratio (Figure 2C; *P*_ANOVA_ = 0.027), which increased significantly in the older mice (R^2^ = 0.382, *p* = 0.008). In parallel, levels of 8-oxodG increased with age (Figure 3A; R^2^ = 0.854, *p* = 0.025). Notably, higher 5hmC/5mC ratios were significantly associated with higher levels of 8-oxodG (R^2^ = 0.785, *p* = 0.046).

Methylation of BER-related gene promotors tended to increase with age, especially in the oldest mice (28 months) (Figure 3B). Methylation of the *Ogg1* promotor (averaged across 27 CpG-sites; Figure S1) increased significantly with age (*P*_ANOVA_ = 0.026; R^2^ = 0.416, *p* = 0.005), with the highest effect observed in 28-month-old mice (*p* = 0.015 vs. three-month-old mice). The average methylation level in the *Xrcc1* promotor was also significantly affected by ageing (*P*_ANOVA_ = 0.023; R^2^ = 0.233, *p* = 0.031): methylation levels in 28-month-old mice were increased compared with three-month-old mice (*P*_ANOVA_ = 0.041). Methylation of four specific CpG-sites in the *Xrcc1* promotor was affected differentially by age (Figure S2). Averaged across 12 CpG-sites, *Neil1* promotor methylation was not significantly affected by age, but methylation of three individual CpG-sites was modulated during ageing (Figure S3). Similarly, *Mutyh* average promotor methylation was not affected by age, but methylation at CpG-site 3 was significantly decreased (Figure S4; *P*_ANOVA_ = 0.014). See Supplementary Materials Figures S1–S4 for details of the individual CpG sites analysed and related TF binding sites for these genes.

### 3.2. Effect of Ageing on Gene Expression in Mouse Brain

For the ease of comparison, gene expression was expressed as the fold change (calculated as log2 of RQ values) compared with expression in three-month-old mice. In line with our hypothesis, there was an overall decrease in BER-related gene expression with age (Figure 3C). This trend was not statistically significant, except for *Ogg1*, where expression decreased significantly with age (*P*_ANOVA_ < 0.001; *P*_(24 vs. 3 months)_ < 0.001; *P*_(28 vs. 3 months)_ < 0.001; and R^2^ = 0.692, *p* < 0.001). This expression change correlated inversely with the average *Ogg1* promoter methylation (Figure S5A; R^2^ = 0.320, *p* = 0.018). Although *Neil1* expression was not significantly affected by age (*P*_ANOVA_ = 0.080), there was a significant inverse correlation between *Neil1* expression and methylation levels at CpG-site 2 (Figure S5B; R^2^ = 0.545, *p* = 0.001). No correlations were observed between gene expression and methylation for *Xrcc1* and *Mutyh*.

### 3.3. Phenotypic Effects in the Ageing Brain

BER-related incision activity in the brain decreased significantly with age (Figure 3D; *P*_ANOVA_ = 0.021). Although not statistically significant, a trend of lower BER activity with decreasing levels of *Ogg1* expression was observed (Figure S6A; R^2^ = 0.173, *p* = 0.068), which seemed to result in higher levels of 8-oxodG lesions (Figure S6B; R^2^ = 0.149, *p* = 0.156). In addition, we observed weak associations between these lower levels of BER-related incision activity and 5hmC levels (Figure S6C; R^2^ = 0.229, *p* = 0.052), as well as 5hmC/5mC ratios (Figure S6D; R^2^ = 0.210, *p* = 0.064).

### 3.4. Involvement of Tet Enzymes and Methyl-CpG Binding Proteins

**Figure 4:**
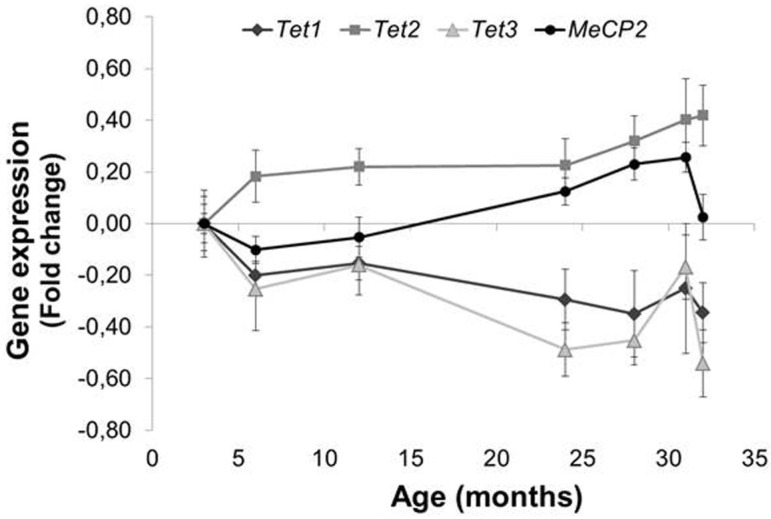
Expression of *Tet1–3* and *Mecp2* with ageing in mouse brain (* *p* = 0.050, ** *p* < 0.040, *** *p* = 0.026). Data are presented as the mean values (*n* = 3–4), and bars indicate standard errors of the mean.

To investigate the involvement of TET enzymes and methyl-CpG binding protein 2 (MECP2) in the effect of ageing on genomic DNA methylation, gene-specific methylation and gene expression, we measured the expression of *Tet1–3* and *Mecp2* genes (Figure 4). *Tet2* (R^2^ = 0.332, *p* = 0.002), and *Mecp2* (*P*_ANOVA_ = 0.003, *P*_(28 vs. 3 months)_ = 0.050, *P*_(31 vs. 3 months)_ = 0.039; R^2^ = 0.241, *p* = 0.011) expression increased significantly with age, while *Tet1* (R^2^ = 0.154, *p* = 0.047) and *Tet3* (*P*_ANOVA_ = 0.033; *P*_(24 vs. 3 months)_ = 0.032, *P*_(28 vs. 3 months)_ = 0.050, *P*_(32 vs. 3 months)_ = 0.026; R^2^ = 0.228, *p* = 0.014) expression decreased with age.

There was an inverse correlation between 5mC levels and *Tet2* expression (Figure S7B; R^2^ = 0.327, *P*_ANOVA_ = 0.016) and lower levels of 5hmC with increasing *Tet1* expression (Figure S7A; R^2^ = 0.444, *P*_ANOVA_ = 0.003). Increased levels of *Ogg1* promotor methylation correlated significantly with increased *Mecp2* expression (Figure S7C; R^2^ = 0.245, *p* = 0.043). Interestingly, *Mecp2* expression also correlated inversely with *Ogg1* expression (Figure S7D; R^2^ = 0.546, *p* < 0.001).

## 4. Discussion

Several lines of evidence link ageing and genome maintenance pathways. Accelerated ageing is observed in mice defective in DNA repair pathways [39,40], and DNA repair deficiency in mature neural tissue has been linked with ageing and common neurodegenerative diseases. For example, certain forms of ataxia are caused by mutations in the BER-associated gene *APTX*, and mutations in NER-related genes (*XPA-XPG*) result in xeroderma pigmentosum, both syndromes that are accompanied by higher rates of neurodegeneration [30]. Such syndromes, which exhibit signs of premature ageing, have been very important in identifying molecular mechanisms that contribute to physiological ageing.

To date, there have been some descriptive studies of epigenetic changes in the ageing brain of Alzheimer patients, but little is known about the role of epigenetic processes in causing the cellular dysfunction, which is characteristic of neurological disorders [41,42]. The study of young versus older monozygotic twins has shown that there are greater inter-twin differences in DNA methylation in older twins, which were associated with greater inter-twin diversity in gene expression profiles [25]. Few studies have investigated promoter-specific methylation of DNA repair-related genes [13,14,15,17,26,27,28], but, for example, Agrelo et al. [28] found that the gene encoding the WRN protein, a RecQ helicase, which is involved in DNA repair, is repressed by promoter hypermethylation in human cancer.

### 4.1. Age-Related Increase in 5hmC, a Result of TET2 or Decreased BER?

In the current study, we did not observe statistically-significant changes in global 5mC levels, though there was a trend of a decline with age in mouse brain. This trend can be confirmed by previous reports of decreased genomic methylation with time observed in cultured cells and with increasing age in tissues from fish, rats, mice and humans (reviewed by [43]). Interestingly the ratio of 5hmC/5mC increased with ageing, which could be a result of increased oxidative stress during ageing (Figure 1). Indeed, levels of 5hmC have previously been reported to increase with age in the brain in animals [23,44] and humans [45], but the specific molecular role of 5hmC is still unclear. Since 5hmC is uniquely enriched in the brain, it is believed to function independently from 5mC and may play a role in mental health and disease [46].

The conversion of 5mC to 5hmC, as an intermediate in the demethylation process, is believed to occur via TET enzyme activity [46]. Interestingly, absolute *Tet1* and *Tet2* expression levels were 4–8-times higher than *Tet3* expression levels (data not shown), which has been reported before in adult cells [47]: *Tet1* and *Tet2* are believed to be important in maintaining pluripotency and adult neurogenesis, while *Tet3* is associated with cell differentiation and mainly involved in pre-natal development [48,49]. In addition, TET3 was reported to mediate increased gene expression that was associated with rapid behavioural adaptation, while TET1 was observed to alter 5hmC patterns during adaptation to longer term stressful environmental exposures (reviewed by Madrid et al., 2016 [46]). Furthermore, TET1, but not TET2 or TET3, may be involved in 5hmC production, which appears to be essential for Purkinje cell viability and the prevention of ataxia-telangiectasia-like symptoms in mice (reviewed by Madrid et al., 2016 [46]).

In the present study, lower 5mC levels were observed with increasing *Tet2* expression, while higher *Tet1* expression seemed to correlate with lower levels of 5hmC. In general, TET enzymes are the major enzymes catalysing conversion of 5mC to 5hmC, and thus, higher levels of 5hmC would be expected to correlate with increased *Tet1* expression, as was the case for *Tet2*. However, TET enzymes also convert 5hmC to 5-formylcytosine (5fC) and 5-carboxylcytosine (5caC) [22,50,51], which could explain the reduced levels of 5hmC observed in our study with increasing *Tet1* expression. In our current study, *Tet1* expression decreased with ageing and can therefore not explain the accumulation of 5hmC with ageing. However, *Tet2* has recently been shown to catalyse the stepwise oxidation of 5mC oxidation and is able to generate 5fC and 5caC from a single encounter with 5mC [51]. However, stalling at 5hmC was observed rather than progression to 5fC and 5caC. In addition, TET2 induction in vitro resulted in increased levels of 5hmC [52]. Although TET2 has been studied most extensively in various types of leukaemia [49], in the current study, *Tet2* expression increased with ageing and was inversely correlated with 5mC levels, which suggests that *Tet2* might be responsible for the age-related accumulation of 5hmC.

Alternatively, the conversion of 5hmC to cytosine may occur via deamination to 5-hydroxymethyluracil (5hmU), resulting in 5hmU-G mismatches that can be excised by BER DNA glycosylases, such as thymine DNA glycosylase (TDG) and NEIL1 [49,53]. Although, *Neil1* expression decreased with age while 5hmC levels increased, we did not observe direct significant associations between DNA glycosylases and 5hmC. In addition, the increased 5hmC levels and 5hmC/5mC ratios with age were significantly associated with lower BER activity, which implies that the higher levels of 5hmC in the ageing brain can be explained, at least partly, by the decreased BER-related incision activity observed in the oldest mice. Recently, in TDG-/- cells, TET2-induced 5-hmC accumulation was observed to result in GC > AT transitions [52], suggesting a mutagenic potential of 5-hmC metabolites if not removed/repaired and which may increase the risk of developing neurological disorders.

### 4.2. Epigenetic Regulation of Ogg1 Plays a Role in Age-Related Decline in DNA Repair

Interestingly, age-related global DNA demethylation has been reported to occur concomitantly with hypermethylation of specific CpG sites in the genome [44,54]. Of particular relevance for the development of age-related disease, site-specific hypermethylation of promoter regions, and transcriptional silencing, of tumour-suppressor genes can occur during aging [44]. Indeed, when using pyrosequencing to quantify methylation at CpG sites in TF binding sites that can influence promoter function, we observed significantly increased methylation of the BER-related *Ogg1* gene promoter with ageing. In addition, and possibly as a consequence, *Ogg1* expression decreased significantly in the oldest mice (Figure 3), and a significant inverse correlation between *Ogg1* expression and promotor methylation was observed (Figure S5). The lack of significant associations between increased promotor methylation and reduced expression for the other BER-related genes could be explained by the fact that bisulphite sequencing does not discriminate between 5mC and 5hmC (reviewed by [55]). Thus, increased methylation levels might be explained by increased 5hmC levels, which may inhibit the binding of methyl-CpG binding proteins and thereby counteract transcriptional repression of 5mC [23,47,56].

Multiple linear regression analysis revealed an inverse correlation between *Neil1* expression and methylation levels of CpG-site 2 located in the binding sites of STAF (or zinc finger protein 143 (ZNF143)) and ZIC2 (Figure S2; CpG-site 2). Enhanced expression of ZNF143, a human homolog of *Xenopus* transcriptional activator Staf, occurs in response to treatment with DNA-damaging agents [57] and induces the expression of DNA repair genes, including the BER-related gene *Fen-1*. ZIC2 activates the transcription of several genes and plays a crucial role in brain development [58].

Our observation of an inverse correlation between *Mecp2* and *Ogg1* expression is further evidence for the epigenetic regulation of this gene (Figure S6). MeCP2 is a methyl-CpG binding protein that can suppress transcription [59]. In the adult brain, MeCP2 binds to methylated DNA and plays a crucial role in normal brain functioning.

In line with our hypothesis, the hypermethylation and decreased gene expression of BER-related genes, especially *Ogg1*, with age was associated with reduced BER-related incision activity. We recently confirmed this association in an independent ageing mice group, where we observed a 43% decrease in *Ogg1* expression and 20% decrease in BER activity in association with increased *Ogg1* promotor methylation in the brain [60]. Several studies have reported lower DNA repair activity in older animals [11,61,62], although others have reported conflicting results [63,64,65]. The lack of direct statistically-significant associations between the expression of the BER-related genes and BER-related incision activity can be explained by the fact that studying gene expression does not necessarily give any indication of enzyme activity. Nonetheless, the age-related decline in BER activity that we observed was paralleled by a significant increase in 8-oxodG levels. This confirms earlier reports of the accumulation of oxidative lesions with ageing [66], which may contribute to the development of a senescent phenotype [67] and increase the risk of neurodegenerative diseases.

### 4.3. Concluding Remarks

We acknowledge the limitation of the size of this study, i.e., the relatively small numbers of mice at each time point (3–4 mice), which, in some cases, may have led to high variability and which may have reduced our ability to detect statistically-significant effects of ageing. Therefore, we are cautious to extrapolate the weak associations observed between BER and 5hmC or 8-oxodG levels. In particular, note that we analysed brain tissue from the oldest mice (31 and 32 months) for *Tet* and *Mecp2* expression only. Furthermore, the brain has a heterogenic mix of cell types with different ratios of neurons to glia cells in the various brain regions. There is evidence that different brain regions have distinct methylation profiles and may respond differently to ageing, which may influence the development of neurodegenerative diseases. Recent studies have indicated a potential role for 5hmC in various neurodegenerative diseases [46], so it will be important to investigate age-related changes in DNA methylation patterns in the various brain regions. Indeed, in an earlier study of brain from six-month-old mice, we reported region-specific patterns of 5mC and 5hmC in the cortex and cerebellum [68]. In addition, we observed differential responses in 5mC and the 5hmC/5mC ratio in these two brain regions following dietary intervention [68]. Although overall 5hmC levels have been reported to increase with age in the brain, depletion of 5hmC was found in the hippocampus, cerebellum and entorhinal cortex of patients suffering from Alzheimer’s disease (AD) [46], while enrichment of 5hmC in the frontal and mid-temporal gyrus was positively correlated with the hallmarks of AD. A recent investigation of the genome-wide distribution of 5hmC in a mouse model of Huntington’s disease (HD) found reduced levels of 5hmC in the mouse striatum and cortex tissues.

In terms of gene-specific methylation profiles, age-related DNA methylation changes are most often observed in CpG islands (as studied in the gene promotors of BER-related genes in the current study), while tissue-specific differences are observed more frequently outside those sites [54]. Future studies of the mechanisms underlying these marked effects of ageing on brain function could also include other genomic domains, e.g., CpG island shores, which are susceptible to altered methylation in response to environmental exposures and which may be important in regulating expression of the corresponding genes. A number of reports show that DNA methylation at intragenic regions (reviewed in Kulis et al., 2013 [69]), CpG island shores [70], partially-methylated domains [71] or long hypomethylated domains [72,73] can influence gene expression. In addition, it may be informative to investigate gene-specific 5hmC and 5mC levels, since *Tet1*-assisted bisulphite sequencing has become an established method for 5hmC detection [46]. Moreover, it will be important to broaden the enquiry to include other epigenetic marks and post-translational modifications, since these work together in a coordinated manner to regulate gene expression.

In addition to the investigation of different brain regions, it would be interesting to investigate the effects of ageing on specific sub-cellular fractions. Increased levels of 8-oxodG lesions during ageing have been reported for nuclear DNA (nDNA) and to a higher extent for mitochondrial DNA (mtDNA). Indeed, the degree of mtDNA oxidative damage in neuronal tissue appears to be inversely related to the maximum life span potential in mammals (reviewed by Gredilla et al., 2010 [74]). Since *Ogg1* is one of the most extensively-investigated mitochondrial DNA glycosylases and recent reports also show the presence of *Neil1* in brain mitochondria (reviewed by Gredilla et al., 2010 [74]), changes in BER-related gene methylation and expression may have a bigger impact on mitochondrial than on nuclear DNA damage and function and, subsequently, on brain ageing. Interestingly, altered mtDNA methylation profiles in human brain have recently been linked to AD and Parkinson’s disease [75]. Although, quantification of mtDNA methylation is still challenging [76], immunoprecipitation methods (e.g., methylated DNA immunoprecipitation (MeDIP)) in combination with microarray hybridization, as well as mass spectrometry-based analysis have been proven very useful in the detection of mtDNA methylation (reviewed by Castegna et al., 2015 [77]).

Although, an increasing number of studies focus on elucidating the molecular events that lead from accumulation of DNA damage, to loss of cellular function and, ultimately, neurodegeneration, further studies are needed to understand the molecular mechanisms underlying age-related changes in DNA repair capacity. The current study helps to solve part of the puzzle and provides evidence that epigenetic mechanisms, i.e., increased *Ogg1* promoter methylation and the involvement of *Tet* enzymes and *Mecp2*, may affect gene expression in the ageing mammalian brain, which could impact the capacity for neuronal DNA repair. Overall, our data suggest that the accelerated accumulation of oxidative DNA damage may be mediated by epigenetic dysregulation of BER activity, causing genome instability and increasing the risk of age-related degenerative neurological diseases.

## Figures and Tables

**Table 1:** Overview of primers and sequences to run pyrosequencing to study gene-specific methylation.

Gene	Amplicon	PCR Primers	Annealing temperature (°C)	Product (bp)	Sequencing primers	Sequence to run on pyrosequencer	Length (bp)
*Ogg1*	1	Fw: 5′-GGTTTATTTTTTGAGATAGA-3′	43	134	5′-TTTAGTTAAGTTTTAAA-3′	C/TGTGTTTTTC/TGTTTTTGTTTATC/TGAGTTTTGGGAC/TGATC/ TGGTGTGTATTATTAC/TGTTTC/TG	60
		Rev: 5′-BIO-ACTAAAACCACATCATTA-3′					
	2	Fw: 5′-GTAGGTTTTGAGATTGTAT-3′	43	184	5′-GAAAGTTTTGAAATGGTAGA-3′	GTG/TGGGTTTTTGGTAGTTAATG/TGTTAAGTAGC/TGAGGTTAGTAGGTT AATC/TGTTTTTATTTTATAGGTTC/TGTTATTTC/TG	79
		Rev: 5′-BIO-ATTTAACCCTAAAAATAAC-3′					
*Neil1*	1	Fw: 5′-TGAGGTAGTAGTTAGTAAGG-3′	52	220	5′-GTAGTTAGTAAGGGGTTAAT-3′	TTTAGTAGTTTGTC/TGAATTTTAGAGTAC/TGTTGGG	34
		Rev: 5′-BIO-ACTCTACTCACAATTCTTT-3′			5′-GAATGGAGTTTTTTATTTATGA-3′	GAATTTC/TGGGTGTTGGGTAACTTTTGGACTAGTC/TGC/ TGTAATTC/TGGAGGTGAC/TGAA	55
	2	Fw: 5′-AGAATTGTGAGTAGAGTTTTGT-3′	52	186	5′-GTTTTAGTTATTTTAGATTATA-3′	C/TGTTAGTAGTC/TGGAAAC/TGGC/TGTTGTGTAGAGTTATAAG TAGTTGTATGC/TGAGG	53
		Rev: 5′-BIO-ATCTTAAATCCCCAAAAATTA-3′					
*Mutyh*	1	Fw: 5′-GGATGGTTATAGAAGTTTAAG-3′	46.6	164	5′-GTTTTAGTTATTTTAGATTATA-3′	ATTTTTAGTGTGTAGC/TGC/TGTGTAATTGTAAAATTC/TG	36
		Rev: 5′-BIO-TCACTACTCCACTCTACAA-3′					
*Xrcc1*	1	Fw: 5′-AGGTTTTAGGAAATTTTTAGTT-3′	50	228	5′-TTTAATGATTAGGGTAAA-3′	TTATAC/TGTAGGATTTAATTATTGAGGTC/TGTTTTTGTTGT TAGGTTTT AGGAGTC/TGAGTTTTTAG/TG	67
		Rev: 5′-BIO-CCCTTAACAACAAACATTC-3′					
	2	Fw: 5′-TGTTTGTTGTTAAGGGAATT-3′	50	328	5′-GGAGAGGTTTAATYGAGTAT-3′	GC/TGTAGTGTTGAC/TGTGTGC/TGTC/TGGC/TGC/TGTC/TGC/TGGTT TGAAAGGTTC/TGAGTTTTGC/TGC/TGTTTGC/TGT	65
		Rev: 5′-BIO-CTCAAAAAACCCCTATCT-3′			5′-GGGGTTTTTTYGGAGTTGTAA-3′	TTTTTTTTTTTTTATTTTTTTGGAC/TGGTC/TGGGC/TGTTTAC/TGGGC/TG TGGATATGTC/TGGAGATTAGTTTTC/TGTTAC/TGTC/TGT	76

BIO = indicates the biotin label on the reverse primers; Y = indicates the presence of an internal C/T wobble in the primer with a non-defined ratio.

**Table 2:** Overview qPCR primers to study gene expression.

	Primer set 1		Primer set 2
Gene	q-PCR primers	Gene	q-PCR primers
*Ogg1*	Fw: 5′-TGGCTTCCCAAACCTCCAT-3′	*Mecp2*	Fw: 5′-GAGGAGGCGAGGAGGAGAGA-3′
	Rev: 5′-GGCCCAACTTCCTCAGGTG-3′		Rev: 5′-AACTTCAGTGGCTTGTCTCTGAGG-3′
*Neil1*	Fw: 5′-GACCCTGAGCCAGAAGATCAG-3′	*Tet1*	Fw: 5′-CCATTCTCACAAGGACATTCACA-3′
	Rev: 5′-AGCTGTGTCTCCTGTGACTT-3′		Rev: 5′-GCAGGACGTGGAGTTGTTCA-3′
*Mutyh*	Fw: 5′-CTGTCTCCCCATATCATCTCTT-3′	*Tet2*	Fw: 5′-GCCATTCTCAGGAGTCACTGC-3′
	Rev: 5′-TCACGCTTCTCTTGGTCATAC-3′		Rev: 5′-ACTTCTCGATTGTCTTCTCTATTGAGG-3′
*Xrrc1*	Fw: 5′-CTTCTCAAGGCGGACACTTA-3′	*Tet3*	Fw: 5′-GGTCACAGCCTGCATGGACT-3′
	Rev: 5′-ATCTGCTCCTCCTTCTCCAA-3′		Rev: 5′-AGCGATTGTCTTCCTTGGTCAG-3′:
*B2m*	Fw: 5′-ATGCTGAAGAACGGGAAAAAAA-3′	*Atp5b*	Fw: 5′-GGCCAAGATGTCCTGCTGTT-3′
	Rev: 5′-CAGTGTGAGCCAGGATATAGAA-3′		Rev: 5′-AACTTTGGCATTGTGGAAGG-3′
*Hprt*	Fw: 5′-AGGAGAGAAAGATGTGATTGATATT-3′	*Gapdh*	Fw: 5′-AACTTTGGCATTGTGGAAGG-3′
	Rev: 5′-TCCACTGAGCAAAACCTCTT-3′		Rev: 5′-ATGCAGGGATGATGTTCTGG-3′

## Supplementary Materials

The following are available online at www.mdpi.com/2073-4425/8/2/75/s1: Figure S1: Overview of the *Ogg1* sequence. Figure S2: Overview of the *Neil1* sequence. Figure S3: Overview of the *Mutyh* sequence. Figure S4: Overview of the *Xrcc1* sequence. Figure S5: Correlation plots between gene expression and DNA methylation profiles. Figure S6: Correlation plots with BER-related incision activity. Figure S7: Linear regression plots.

## Abbreviations

The following abbreviations are used in this manuscript:

5caC	5-carboxylcytosine
5fC	5-formylcytosine
5hmC	5-hydroxymethycytosine
5mC	5-methylcytosine
8-oxodG	8-oxo-7,8-dihydro-2′-deoxyguanosine
BER	base excision repair
*Mecp2*	methyl-CpG binding protein 2
*Mutyh*	mutY DNA glycosylase
nDNA	nuclear DNA
NER	nucleotide excision repair
*Neil1*	Nei-like DNA glycosylase 1
mtDNA	mitochondrial DNA
*Ogg1*	DNA glycosylase oxoguanosine 1
ROS	reactive oxygen species;
TDG	thymine DNA glycosylase
TEMPO	2,2,6,6-tetramethylpiperidine-*N*-oxyl
TET	ten-eleven translocation enzymes
TF	transcription factors
*Xrcc1*	X-ray repair cross-complementing protein 1

## References

[1] Cooke M.S., Evans M.D., Dizdaroglu M., Lunec J. (2003). Oxidative DNA damage: Mechanisms, mutation, and disease. FASEB J..

[2] Lopez-Otin C., Blasco M.A., Partridge L., Serrano M., Kroemer G. (2013). The hallmarks of aging. Cell.

[3] Langie S.A.S., Lara J., Mathers J.C. (2012). Early determinants of the ageing trajectory. Best Pract. Res. Clin. Endocrinol. Metab..

[4] Parsons J.L., Zharkov D.O., Dianov G.L. (2005). NEIL1 excises 3′ end proximal oxidative DNA lesions resistant to cleavage by NTH1 and OGG1. Nucleic Acids Res..

[5] Robertson A.B., Klungland A., Rognes T., Leiros I. (2009). DNA repair in mammalian cells: Base excision repair: The long and short of it. Cell. Mol. Life Sci..

[6] Marsin S., Vidal A.E., Sossou M., Menissier-de Murcia J., Le Page F., Boiteux S., de Murcia G., Radicella J.P. (2003). Role of XRCC1 in the coordination and stimulation of oxidative DNA damage repair initiated by the DNA glycosylase hOGG1. J. Biol. Chem..

[7] Osterod M., Hollenbach S., Hengstler J.G., Barnes D.E., Lindahl T., Epe B. (2001). Age-related and tissue-specific accumulation of oxidative DNA base damage in 7,8-dihydro-8-oxoguanine-DNA glycosylase (*Ogg1*) deficient mice. Carcinogenesis.

[8] Chen S.K., Hsieh W.A., Tsai M.H., Chen C.C., Hong A.I., Wei Y.H., Chang W.P. (2003). Age-associated decrease of oxidative repair enzymes, human 8-oxoguanine DNA glycosylases (hOgg1), in human aging. J. Radiat. Res..

[9] Jacob K.D., Noren Hooten N., Tadokoro T., Lohani A., Barnes J., Evans M.K. (2013). Alzheimer’s disease-associated polymorphisms in human OGG1 alter catalytic activity and sensitize cells to DNA damage. Free Radic. Biol. Med..

[10] Mao G., Pan X., Zhu B.B., Zhang Y., Yuan F., Huang J., Lovell M.A., Lee M.P., Markesbery W.R., Li G.M. (2007). Identification and characterization of OGG1 mutations in patients with alzheimer’s disease. Nucleic Acids Res..

[11] Cabelof D.C., Raffoul J.J., Yanamadala S., Ganir C., Guo Z., Heydari A.R. (2002). Attenuation of DNA polymerase beta-dependent base excision repair and increased DMS-induced mutagenicity in aged mice. Mutat. Res..

[12] Noren Hooten N., Fitzpatrick M., Kompaniez K., Jacob K.D., Moore B.R., Nagle J., Barnes J., Lohani A., Evans M.K. (2012). Coordination of DNA repair by NEIL1 and PARP-1: A possible link to aging. Aging.

[13] Esteller M. (2007). Cancer epigenomics: DNA methylomes and histone-modification maps. Nat. Rev. Genet..

[14] Fang M.Z., Chen D., Sun Y., Jin Z., Christman J.K., Yang C.S. (2005). Reversal of hypermethylation and reactivation of *p16^INK4a^*, *RARbeta*, and *MGMT* genes by genistein and other isoflavones from soy. Clin. Cancer Res..

[15] Fang M.Z., Wang Y., Ai N., Hou Z., Sun Y., Lu H., Welsh W., Yang C.S. (2003). Tea polyphenol (−)-epigallocatechin-3-gallate inhibits DNA methyltransferase and reactivates methylation-silenced genes in cancer cell lines. Cancer Res..

[16] Langie S.A., Koppen G., Desaulniers D., Al-Mulla F., Al-Temaimi R., Amedei A., Azqueta A., Bisson W.H., Brown D.G., Brunborg G. (2015). Causes of genome instability: The effect of low dose chemical exposures in modern society. Carcinogenesis.

[17] Langie S.A., Kowalczyk P., Tomaszewski B., Vasilaki A., Maas L.M., Moonen E.J., Palagani A., Godschalk R.W., Tudek B., van Schooten F.J. (2014). Redox and epigenetic regulation of the *APE1* gene in the hippocampus of piglets: The effect of early life exposures. DNA Repair.

[18] Mehler M.F. (2008). Epigenetic principles and mechanisms underlying nervous system functions in health and disease. Prog. Neurobiol..

[19] Jones P.A., Takai D. (2001). The role of DNA methylation in mammalian epigenetics. Science.

[20] Calvanese V., Lara E., Kahn A., Fraga M.F. (2009). The role of epigenetics in aging and age-related diseases. Ageing Res. Rev..

[21] Mathers J.C., Strathdee G., Relton C.L., Herceg Z., Ushijima T. (2010). Induction of epigenetic alterations by dietary and other environmental factors. Advances in Genetics.

[22] Nabel C.S., Kohli R.M. (2011). Molecular biology. Demystifying DNA demethylation. Science.

[23] Van den Hove D.L., Chouliaras L., Rutten B.P. (2012). The role of 5-hydroxymethylcytosine in aging and Alzheimer’s disease: Current status and prospects for future studies. Curr. Alzheimer Res..

[24] Rasmussen K.D., Helin K. (2016). Role of TET enzymes in DNA methylation, development, and cancer. Genes Dev..

[25] Fraga M.F., Ballestar E., Paz M.F., Ropero S., Setien F., Ballestar M.L., Heine-Suner D., Cigudosa J.C., Urioste M., Benitez J. (2005). Epigenetic differences arise during the lifetime of monozygotic twins. Proc. Natl. Acad. Sci. USA.

[26] Arai T., Kasahara I., Sawabe M., Honma N., Aida J., Tabubo K. (2010). Role of methylation of the *hMLH1* gene promoter in the development of gastric and colorectal carcinoma in the elderly. Geriatr. Gerontol. Int..

[27] Wheeler J.M. (2005). Epigenetics, mismatch repair genes and colorectal cancer. Ann. R. Coll. Surg. Engl..

[28] Agrelo R., Cheng W.H., Setien F., Ropero S., Espada J., Fraga M.F., Herranz M., Paz M.F., Sanchez-Cespedes M., Artiga M.J. (2006). Epigenetic inactivation of the premature aging werner syndrome gene in human cancer. Proc. Natl. Acad. Sci. USA.

[29] Butterfield D.A., Reed T., Newman S.F., Sultana R. (2007). Roles of amyloid beta-peptide-associated oxidative stress and brain protein modifications in the pathogenesis of alzheimer’s disease and mild cognitive impairment. Free Radic. Biol. Med..

[30] McKinnon P.J. (2009). DNA repair deficiency and neurological disease. Nat. Rev. Neurosci..

[31] Nouspikel T., Hanawalt P.C. (2003). When parsimony backfires: Neglecting DNA repair may doom neurons in alzheimer’s disease. BioEssays.

[32] Rowlatt C., Chesterman F.C., Sheriff M.U. (1976). Lifespan, age changes and tumour incidence in an ageing C57BL mouse colony. Lab. Anim..

[33] Godschalk R.W., Maas L.M., van Zandwijk N., van’t Veer L.J., Breedijk A., Borm P.J., Verhaert J., Kleinjans J.C., van Schooten F.J. (1998). Differences in aromatic-DNA adduct levels between alveolar macrophages and subpopulations of white blood cells from smokers. Carcinogenesis.

[34] European Standards Committee on Oxidative DNA Damage (2000). Comparison of different methods of measuring 8-oxoguanine as a marker of oxidative DNA damage. Free Radic. Res..

[35] Langie S.A., Kowalczyk P., Tudek B., Zabielski R., Dziaman T., Olinski R., van Schooten F.J., Godschalk R.W. (2010). The effect of oxidative stress on nucleotide-excision repair in colon tissue of newborn piglets. Mutat. Res..

[36] Wang Y., Leung F.C. (2004). An evaluation of new criteria for cpg islands in the human genome as gene markers. Bioinformatics.

[37] Law J.A., Jacobsen S.E. (2010). Establishing, maintaining and modifying DNA methylation patterns in plants and animals. Nat. Rev. Genet..

[38] Langie S.A., Cameron K.M., Waldron K.J., Fletcher K.P., von Zglinicki T., Mathers J.C. (2011). Measuring DNA repair incision activity of mouse tissue extracts towards singlet oxygen-induced DNA damage: A comet-based in vitro repair assay. Mutagenesis.

[39] Borgesius N.Z., de Waard M.C., van der Pluijm I., Omrani A., Zondag G.C., van der Horst G.T., Melton D.W., Hoeijmakers J.H., Jaarsma D., Elgersma Y. (2011). Accelerated age-related cognitive decline and neurodegeneration, caused by deficient DNA repair. J. Neurosci..

[40] De Waard M.C., van der Pluijm I., Zuiderveen Borgesius N., Comley L.H., Haasdijk E.D., Rijksen Y., Ridwan Y., Zondag G., Hoeijmakers J.H., Elgersma Y. (2010). Age-related motor neuron degeneration in DNA repair-deficient Ercc1 mice. Acta Neuropathol..

[41] Mathers J.C., Coxhead J.M., Tyson J. (2007). Nutrition and DNA repair—Potential molecular mechanisms of action. Curr. Cancer Drug Targets.

[42] Zawia N.H., Lahiri D.K., Cardozo-Pelaez F. (2009). Epigenetics, oxidative stress, and alzheimer disease. Free Radic. Biol. Med..

[43] Hochberg Z., Feil R., Constancia M., Fraga M., Junien C., Carel J.C., Boileau P., Le Bouc Y., Deal C.L., Lillycrop K. (2011). Child health, developmental plasticity, and epigenetic programming. Endocr. Rev..

[44] Jung M., Pfeifer G.P. (2015). Aging and DNA methylation. BMC Biol..

[45] Wagner M., Steinbacher J., Kraus T.F., Michalakis S., Hackner B., Pfaffeneder T., Perera A., Muller M., Giese A., Kretzschmar H.A. (2015). Age-dependent levels of 5-methyl-, 5-hydroxymethyl-, and 5-formylcytosine in human and mouse brain tissues. Angew. Chem. Int. Ed. Engl..

[46] Madrid A., Papale L.A., Alisch R.S. (2016). New hope: The emerging role of 5-hydroxymethylcytosine in mental health and disease. Epigenomics.

[47] Munzel M., Globisch D., Carell T. (2011). 5-hydroxymethylcytosine, the sixth base of the genome. Angew. Chem. Int. Ed. Engl..

[48] Kohli R.M., Zhang Y. (2013). TET enzymes, TDG and the dynamics of DNA demethylation. Nature.

[49] Tan L., Shi Y.G. (2012). TET family proteins and 5-hydroxymethylcytosine in development and disease. Development.

[50] Santiago M., Antunes C., Guedes M., Sousa N., Marques C.J. (2014). TET enzymes and DNA hydroxymethylation in neural development and function—How critical are they?. Genomics.

[51] Crawford D.J., Liu M.Y., Nabel C.S., Cao X.J., Garcia B.A., Kohli R.M. (2016). *Tet2* catalyzes stepwise 5-methylcytosine oxidation by an iterative and de novo mechanism. J. Am. Chem. Soc..

[52] Mahfoudhi E., Talhaoui I., Cabagnols X., Della Valle V., Secardin L., Rameau P., Bernard O.A., Ishchenko A.A., Abbes S., Vainchenker W. (2016). *Tet2*-mediated 5-hydroxymethylcytosine induces genetic instability and mutagenesis. DNA Repair.

[53] Meng H., Cao Y., Qin J., Song X., Zhang Q., Shi Y., Cao L. (2015). DNA methylation, its mediators and genome integrity. Int. J. Biol. Sci..

[54] Pal S., Tyler J.K. (2016). Epigenetics and aging. Sci. Adv..

[55] Booth M.J., Branco M.R., Ficz G., Oxley D., Krueger F., Reik W., Balasubramanian S. (2012). Quantitative sequencing of 5-methylcytosine and 5-hydroxymethylcytosine at single-base resolution. Science.

[56] Branco M.R., Ficz G., Reik W. (2012). Uncovering the role of 5-hydroxymethylcytosine in the epigenome. Nature Rev. Genet..

[57] Wakasugi T., Izumi H., Uchiumi T., Suzuki H., Arao T., Nishio K., Kohno K. (2007). ZNF143 interacts with p73 and is involved in cisplatin resistance through the transcriptional regulation of DNA repair genes. Oncogene.

[58] Ishiguro A., Aruga J. (2008). Functional role of Zic2 phosphorylation in transcriptional regulation. FEBS Lett..

[59] Riccio A. (2010). Dynamic epigenetic regulation in neurons: Enzymes, stimuli and signaling pathways. Nat. Neurosci..

[60] Gorniak J., Langie S.A.S., Cameron K., von Zglinicki T., Mathers J.C. (2012). The effect of ageing and short-term dietary restriction on the epigenetic, transcriptomic and phenotypic profile of base excision repair in mouse brain and liver. Proc. Nutr. Soc..

[61] Cabelof D.C., Yanamadala S., Raffoul J.J., Guo Z., Soofi A., Heydari A.R. (2003). Caloric restriction promotes genomic stability by induction of base excision repair and reversal of its age-related decline. DNA Repair.

[62] Imam S.Z., Karahalil B., Hogue B.A., Souza-Pinto N.C., Bohr V.A. (2006). Mitochondrial and nuclear DNA-repair capacity of various brain regions in mouse is altered in an age-dependent manner. Neurobiol. Aging.

[63] Xu G., Herzig M., Rotrekl V., Walter C.A. (2008). Base excision repair, aging and health span. Mech. Ageing Dev..

[64] Rao K.S. (2003). Dietary calorie restriction, DNA-repair and brain aging. Mol. Cell. Biochem..

[65] Gedik C.M., Grant G., Morrice P.C., Wood S.G., Collins A.R. (2005). Effects of age and dietary restriction on oxidative DNA damage, antioxidant protection and DNA repair in rats. Eur. J. Nutr..

[66] Moller P., Lohr M., Folkmann J.K., Mikkelsen L., Loft S. (2010). Aging and oxidatively damaged nuclear DNA in animal organs. Free Radic. Biol. Med..

[67] Jurk D., Wang C., Miwa S., Maddick M., Korolchuk V., Tsolou A., Gonos E.S., Thrasivoulou C., Saffrey M.J., Cameron K. (2012). Postmitotic neurons develop a p21-dependent senescence-like phenotype driven by a DNA damage response. Aging Cell.

[68] Langie S.A., Achterfeldt S., Gorniak J.P., Halley-Hogg K.J., Oxley D., van Schooten F.J., Godschalk R.W., McKay J.A., Mathers J.C. (2013). Maternal folate depletion and high-fat feeding from weaning affects DNA methylation and DNA repair in brain of adult offspring. FASEB J..

[69] Kulis M., Queiros A.C., Beekman R., Martin-Subero J.I. (2013). Intragenic DNA methylation in transcriptional regulation, normal differentiation and cancer. Biochim. Biophys. Acta.

[70] Doi A., Park I.H., Wen B., Murakami P., Aryee M.J., Irizarry R., Herb B., Ladd-Acosta C., Rho J., Loewer S. (2009). Differential methylation of tissue- and cancer-specific cpg island shores distinguishes human induced pluripotent stem cells, embryonic stem cells and fibroblasts. Nat. Genet..

[71] Hon G.C., Hawkins R.D., Caballero O.L., Lo C., Lister R., Pelizzola M., Valsesia A., Ye Z., Kuan S., Edsall L.E. (2012). Global DNA hypomethylation coupled to repressive chromatin domain formation and gene silencing in breast cancer. Genome Res..

[72] Berman B.P., Weisenberger D.J., Aman J.F., Hinoue T., Ramjan Z., Liu Y., Noushmehr H., Lange C.P., van Dijk C.M., Tollenaar R.A. (2011). Regions of focal DNA hypermethylation and long-range hypomethylation in colorectal cancer coincide with nuclear lamina-associated domains. Nat. Genet..

[73] Hansen K.D., Timp W., Bravo H.C., Sabunciyan S., Langmead B., McDonald O.G., Wen B., Wu H., Liu Y., Diep D. (2011). Increased methylation variation in epigenetic domains across cancer types. Nat. Genet..

[74] Gredilla R., Bohr V.A., Stevnsner T. (2010). Mitochondrial DNA repair and association with aging—An update. Exp. Gerontol..

[75] Blanch M., Mosquera J.L., Ansoleaga B., Ferrer I., Barrachina M. (2016). Altered mitochondrial DNA methylation pattern in alzheimer disease-related pathology and in parkinson disease. Am. J. Pathol..

[76] Byun H.M., Barrow T.M. (2015). Analysis of pollutant-induced changes in mitochondrial DNA methylation. Methods Mol. Biol..

[77] Castegna A., Iacobazzi V., Infantino V. (2015). The mitochondrial side of epigenetics. Physiol. Genom..

