# Supplementary material for: The Ageing Brain: Effects on DNA Repair and DNA Methylation in Mice
Source: Genes (Basel). 2017 Feb 17;8(2):75. doi: 10.3390/genes8020075 (PMC5333064; doi:10.3390/genes8020075)
Supplement: Supplementary file 1 [file genes-08-00075-s001.pptx]

## Slide 1
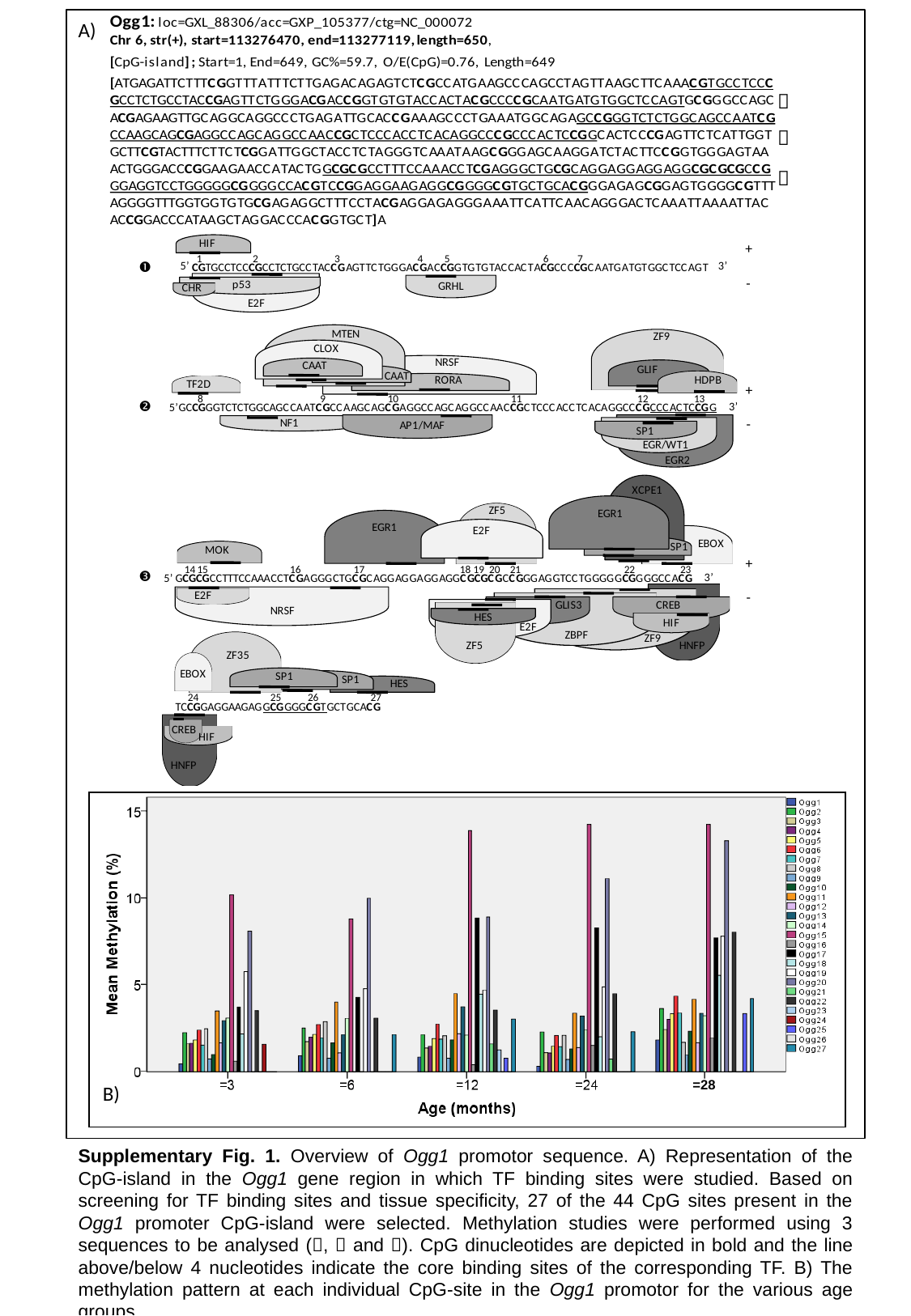

A)



B)
=28
Supplementary Fig. 1. Overview of Ogg1 promotor sequence. A) Representation of the CpG-island in the Ogg1 gene region in which TF binding sites were studied. Based on screening for TF binding sites and tissue specificity, 27 of the 44 CpG sites present in the Ogg1 promoter CpG-island were selected. Methylation studies were performed using 3 sequences to be analysed (,  and ). CpG dinucleotides are depicted in bold and the line above/below 4 nucleotides indicate the core binding sites of the corresponding TF. B) The methylation pattern at each individual CpG-site in the Ogg1 promotor for the various age groups.

## Slide 2
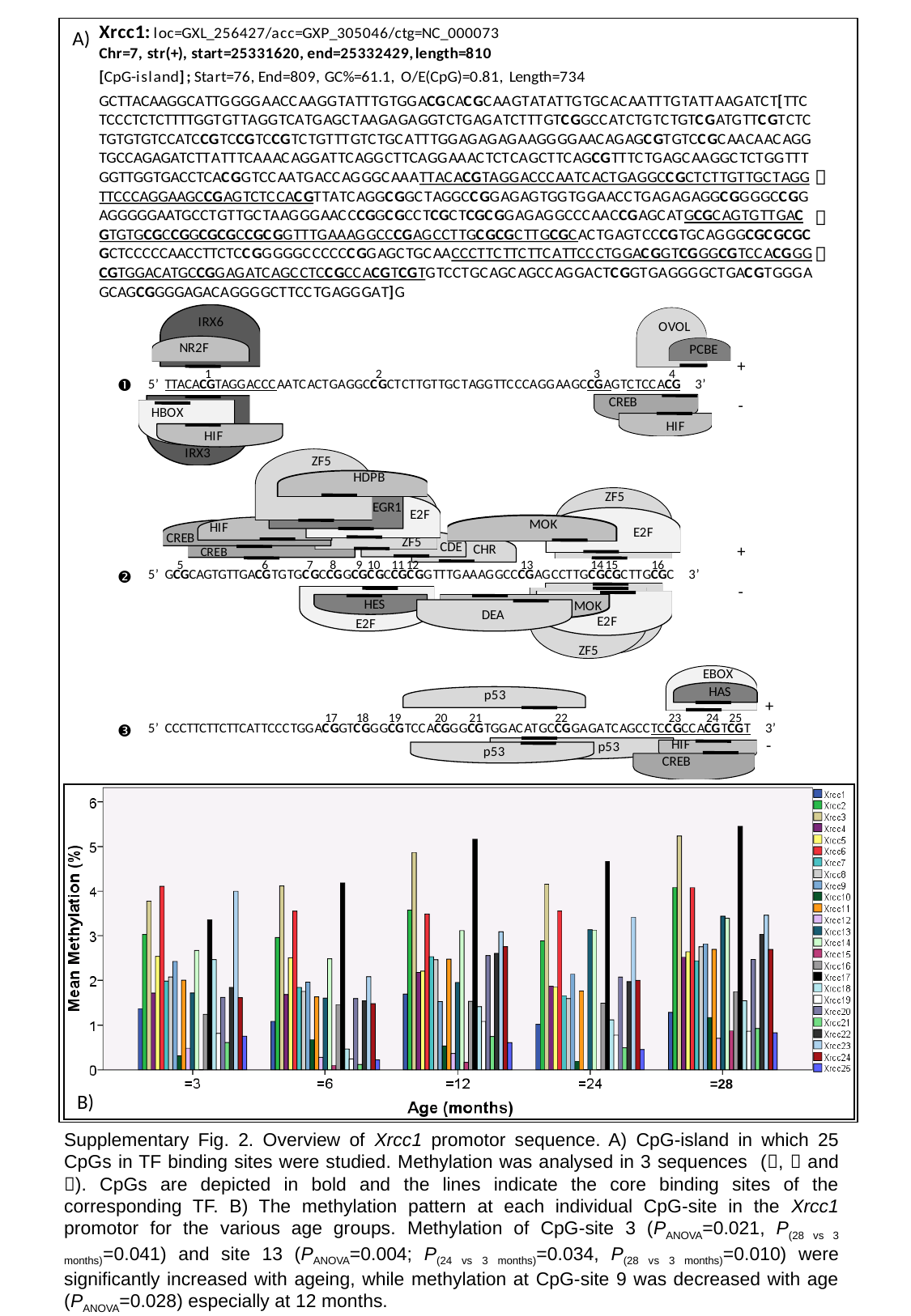

A)



B)
=28
Supplementary Fig. 2. Overview of Xrcc1 promotor sequence. A) CpG-island in which 25 CpGs in TF binding sites were studied. Methylation was analysed in 3 sequences (,  and ). CpGs are depicted in bold and the lines indicate the core binding sites of the corresponding TF. B) The methylation pattern at each individual CpG-site in the Xrcc1 promotor for the various age groups. Methylation of CpG-site 3 (PANOVA=0.021, P(28 vs 3 months)=0.041) and site 13 (PANOVA=0.004; P(24 vs 3 months)=0.034, P(28 vs 3 months)=0.010) were significantly increased with ageing, while methylation at CpG-site 9 was decreased with age (PANOVA=0.028) especially at 12 months.

## Slide 3
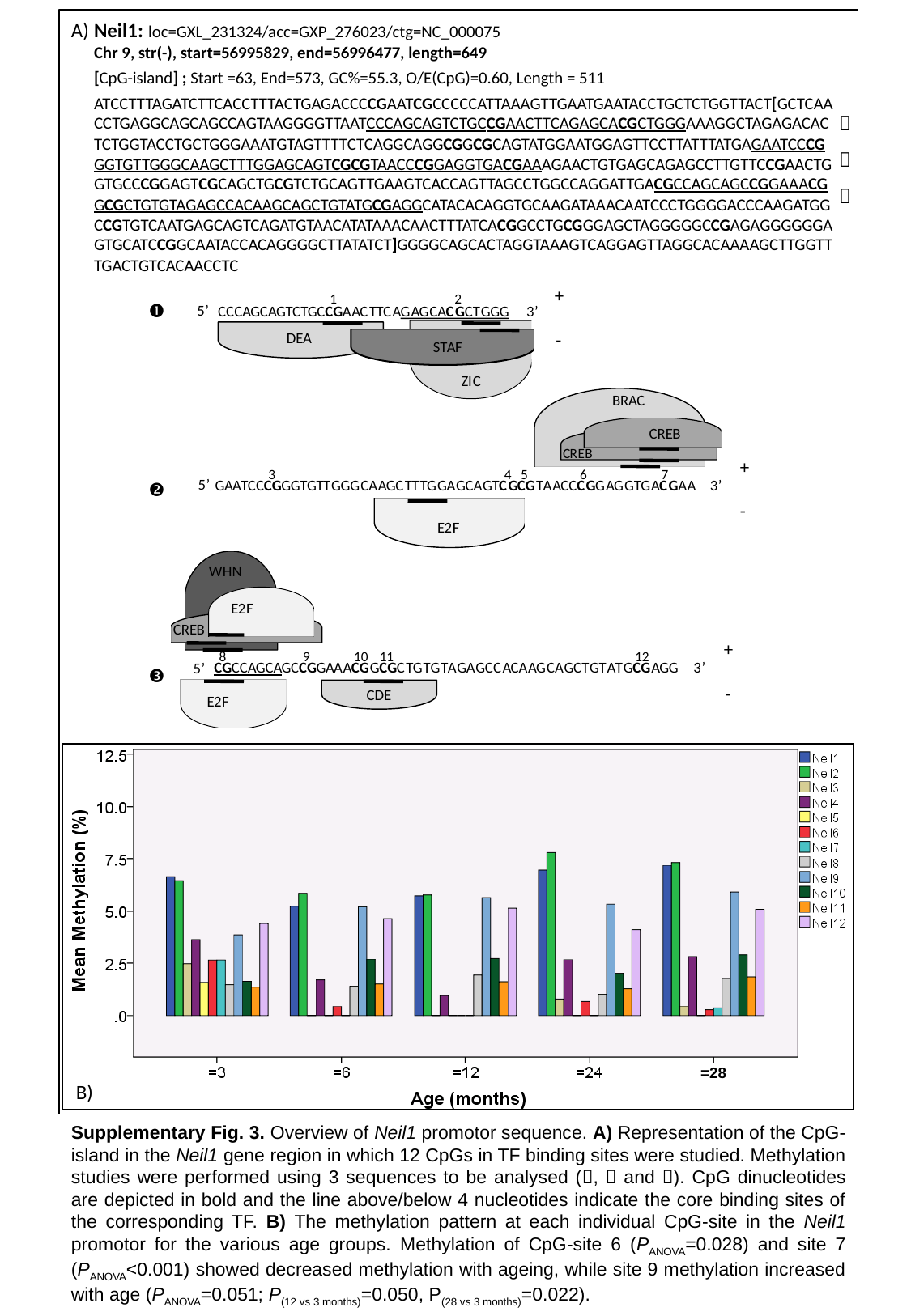

A)
Neil1: loc=GXL_231324/acc=GXP_276023/ctg=NC_000075
Chr 9, str(-), start=56995829, end=56996477, length=649
[CpG-island] ; Start =63, End=573, GC%=55.3, O/E(CpG)=0.60, Length = 511
ATCCTTTAGATCTTCACCTTTACTGAGACCCCGAATCGCCCCCATTAAAGTTGAATGAATACCTGCTCTGGTTACT[GCTCAACCTGAGGCAGCAGCCAGTAAGGGGTTAATCCCAGCAGTCTGCCGAACTTCAGAGCACGCTGGGAAAGGCTAGAGACACTCTGGTACCTGCTGGGAAATGTAGTTTTCTCAGGCAGGCGGCGCAGTATGGAATGGAGTTCCTTATTTATGAGAATCCCGGGTGTTGGGCAAGCTTTGGAGCAGTCGCGTAACCCGGAGGTGACGAAAGAACTGTGAGCAGAGCCTTGTTCCGAACTGGTGCCCGGAGTCGCAGCTGCGTCTGCAGTTGAAGTCACCAGTTAGCCTGGCCAGGATTGACGCCAGCAGCCGGAAACGGCGCTGTGTAGAGCCACAAGCAGCTGTATGCGAGGCATACACAGGTGCAAGATAAACAATCCCTGGGGACCCAAGATGGCCGTGTCAATGAGCAGTCAGATGTAACATATAAACAACTTTATCACGGCCTGCGGGAGCTAGGGGGCCGAGAGGGGGGAGTGCATCCGGCAATACCACAGGGGCTTATATCT]GGGGCAGCACTAGGTAAAGTCAGGAGTTAGGCACAAAAGCTTGGTTTGACTGTCACAACCTC



B)
=28
Supplementary Fig. 3. Overview of Neil1 promotor sequence. A) Representation of the CpG-island in the Neil1 gene region in which 12 CpGs in TF binding sites were studied. Methylation studies were performed using 3 sequences to be analysed (,  and ). CpG dinucleotides are depicted in bold and the line above/below 4 nucleotides indicate the core binding sites of the corresponding TF. B) The methylation pattern at each individual CpG-site in the Neil1 promotor for the various age groups. Methylation of CpG-site 6 (PANOVA=0.028) and site 7 (PANOVA<0.001) showed decreased methylation with ageing, while site 9 methylation increased with age (PANOVA=0.051; P(12 vs 3 months)=0.050, P(28 vs 3 months)=0.022).

## Slide 4
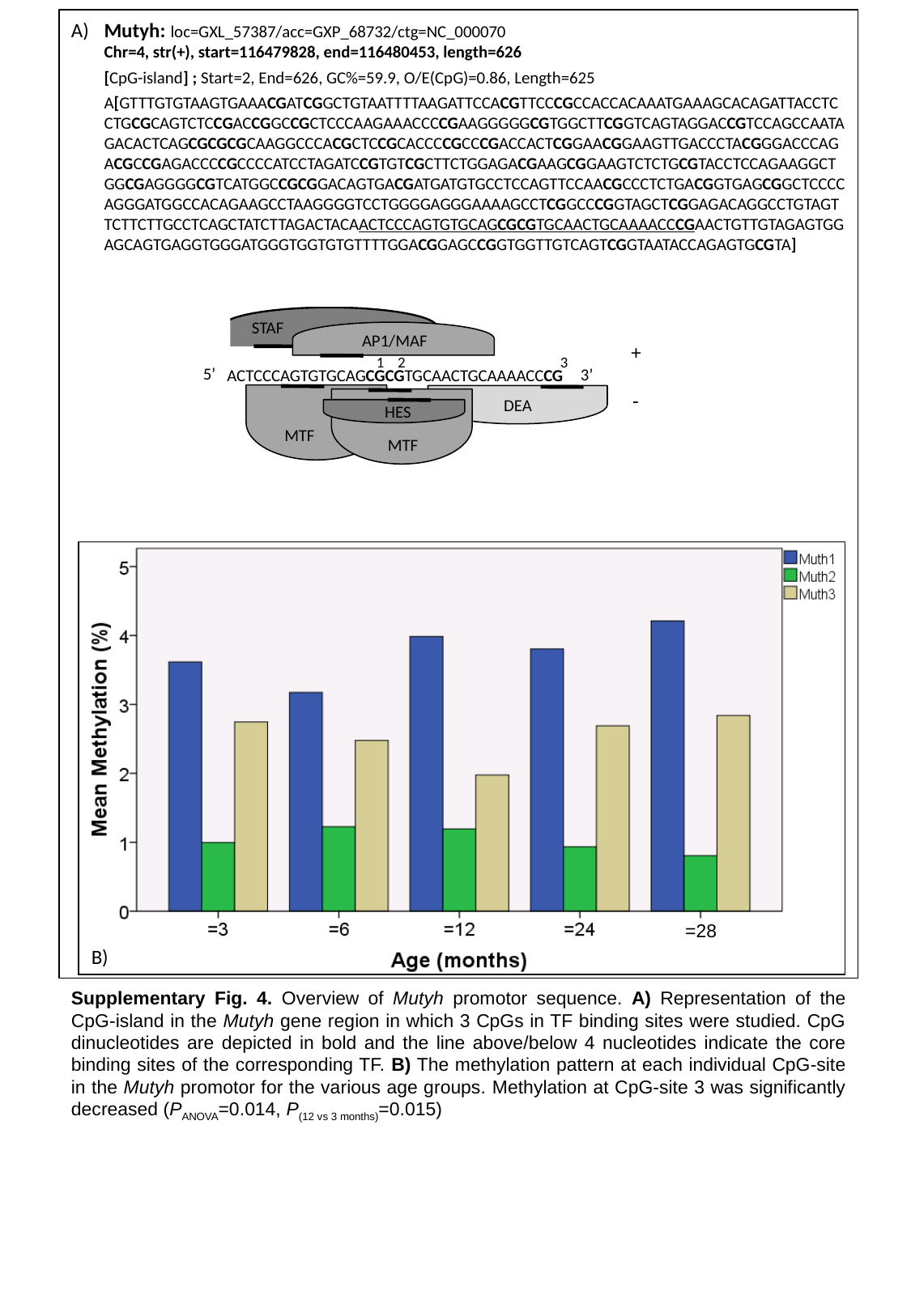

Mutyh: loc=GXL_57387/acc=GXP_68732/ctg=NC_000070
Chr=4, str(+), start=116479828, end=116480453, length=626
[CpG-island] ; Start=2, End=626, GC%=59.9, O/E(CpG)=0.86, Length=625
A[GTTTGTGTAAGTGAAACGATCGGCTGTAATTTTAAGATTCCACGTTCCCGCCACCACAAATGAAAGCACAGATTACCTCCTGCGCAGTCTCCGACCGGCCGCTCCCAAGAAACCCCGAAGGGGGCGTGGCTTCGGTCAGTAGGACCGTCCAGCCAATAGACACTCAGCGCGCGCAAGGCCCACGCTCCGCACCCCGCCCGACCACTCGGAACGGAAGTTGACCCTACGGGACCCAGACGCCGAGACCCCGCCCCATCCTAGATCCGTGTCGCTTCTGGAGACGAAGCGGAAGTCTCTGCGTACCTCCAGAAGGCTGGCGAGGGGCGTCATGGCCGCGGACAGTGACGATGATGTGCCTCCAGTTCCAACGCCCTCTGACGGTGAGCGGCTCCCCAGGGATGGCCACAGAAGCCTAAGGGGTCCTGGGGAGGGAAAAGCCTCGGCCCGGTAGCTCGGAGACAGGCCTGTAGTTCTTCTTGCCTCAGCTATCTTAGACTACAACTCCCAGTGTGCAGCGCGTGCAACTGCAAAACCCGAACTGTTGTAGAGTGGAGCAGTGAGGTGGGATGGGTGGTGTGTTTTGGACGGAGCCGGTGGTTGTCAGTCGGTAATACCAGAGTGCGTA]
A)
STAF
AP1/MAF
+
-
	1 2 3
5’
3’
ACTCCCAGTGTGCAGCGCGTGCAACTGCAAAACCCG
DEA
HES
MTF
MTF
B)
=28
Supplementary Fig. 4. Overview of Mutyh promotor sequence. A) Representation of the CpG-island in the Mutyh gene region in which 3 CpGs in TF binding sites were studied. CpG dinucleotides are depicted in bold and the line above/below 4 nucleotides indicate the core binding sites of the corresponding TF. B) The methylation pattern at each individual CpG-site in the Mutyh promotor for the various age groups. Methylation at CpG-site 3 was significantly decreased (PANOVA=0.014, P(12 vs 3 months)=0.015)

## Slide 5
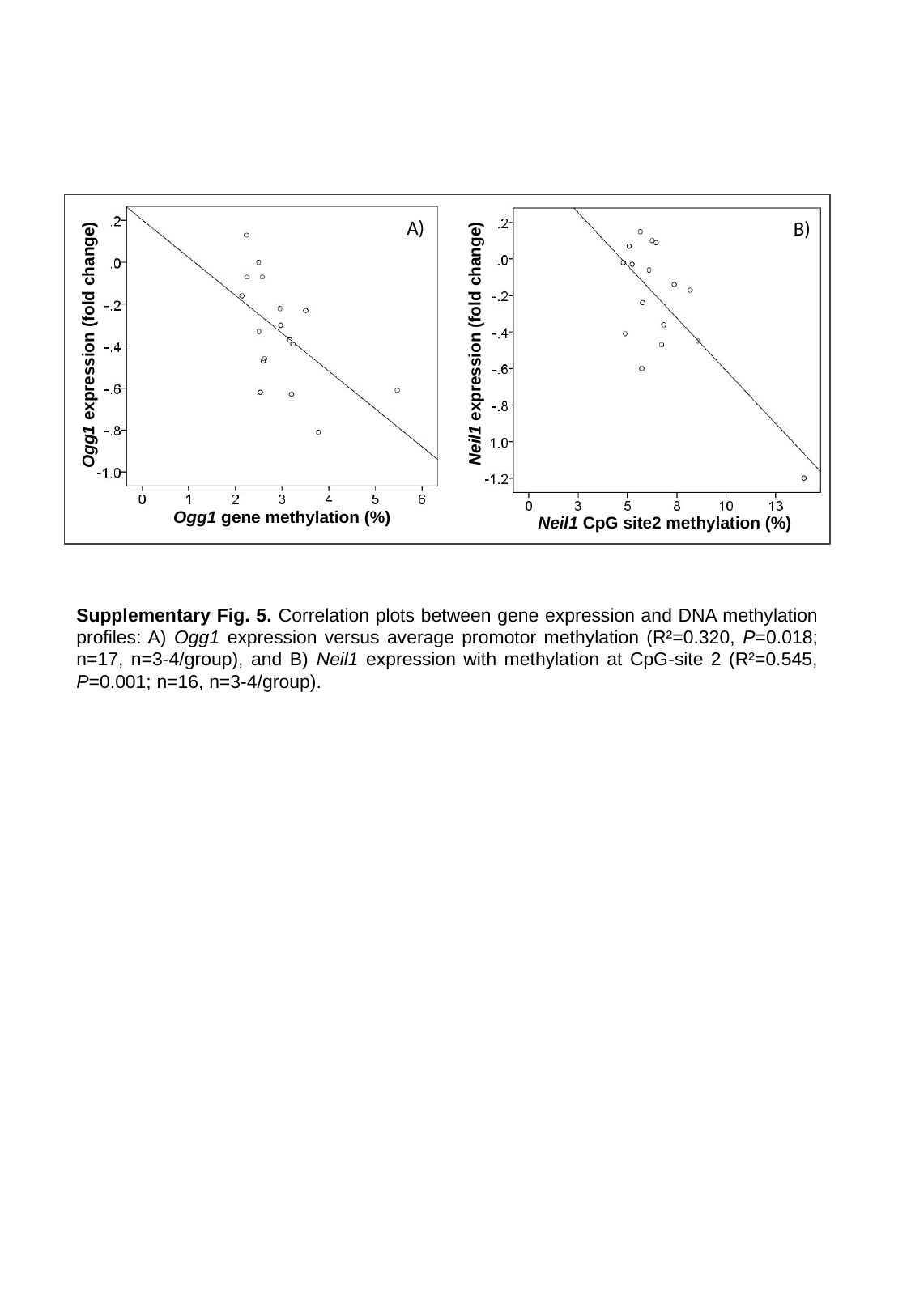

Ogg1 expression (fold change)
Ogg1 gene methylation (%)
Neil1 expression (fold change)
Neil1 CpG site2 methylation (%)
A)
B)
Supplementary Fig. 5. Correlation plots between gene expression and DNA methylation profiles: A) Ogg1 expression versus average promotor methylation (R²=0.320, P=0.018; n=17, n=3-4/group), and B) Neil1 expression with methylation at CpG-site 2 (R²=0.545, P=0.001; n=16, n=3-4/group).

## Slide 6
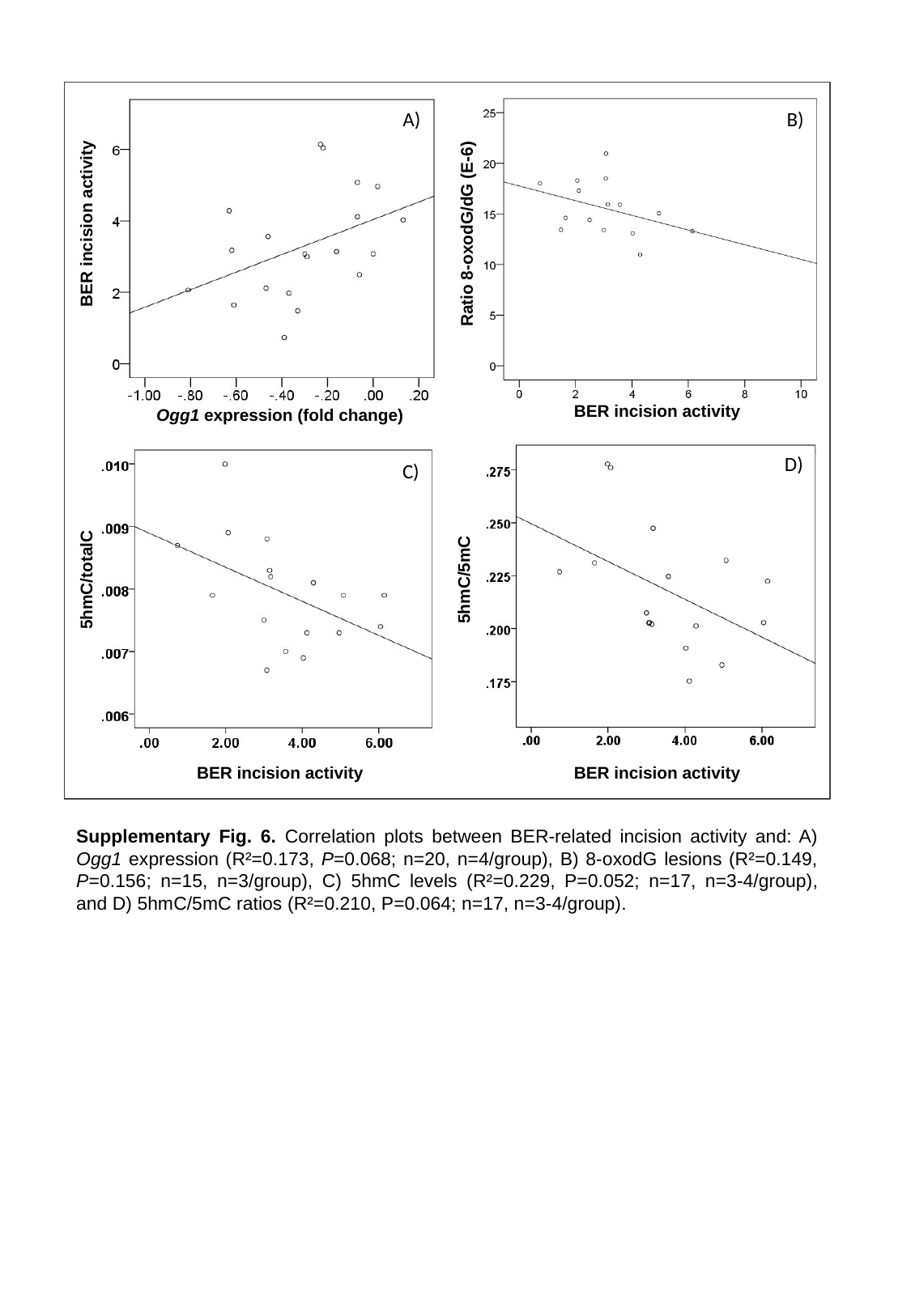

Ratio 8-oxodG/dG (E-6)
BER incision activity
A)
B)
BER incision activity
Ogg1 expression (fold change)
D)
C)
5hmC/totalC
5hmC/5mC
BER incision activity
BER incision activity
Supplementary Fig. 6. Correlation plots between BER-related incision activity and: A) Ogg1 expression (R²=0.173, P=0.068; n=20, n=4/group), B) 8-oxodG lesions (R²=0.149, P=0.156; n=15, n=3/group), C) 5hmC levels (R²=0.229, P=0.052; n=17, n=3-4/group), and D) 5hmC/5mC ratios (R²=0.210, P=0.064; n=17, n=3-4/group).

## Slide 7
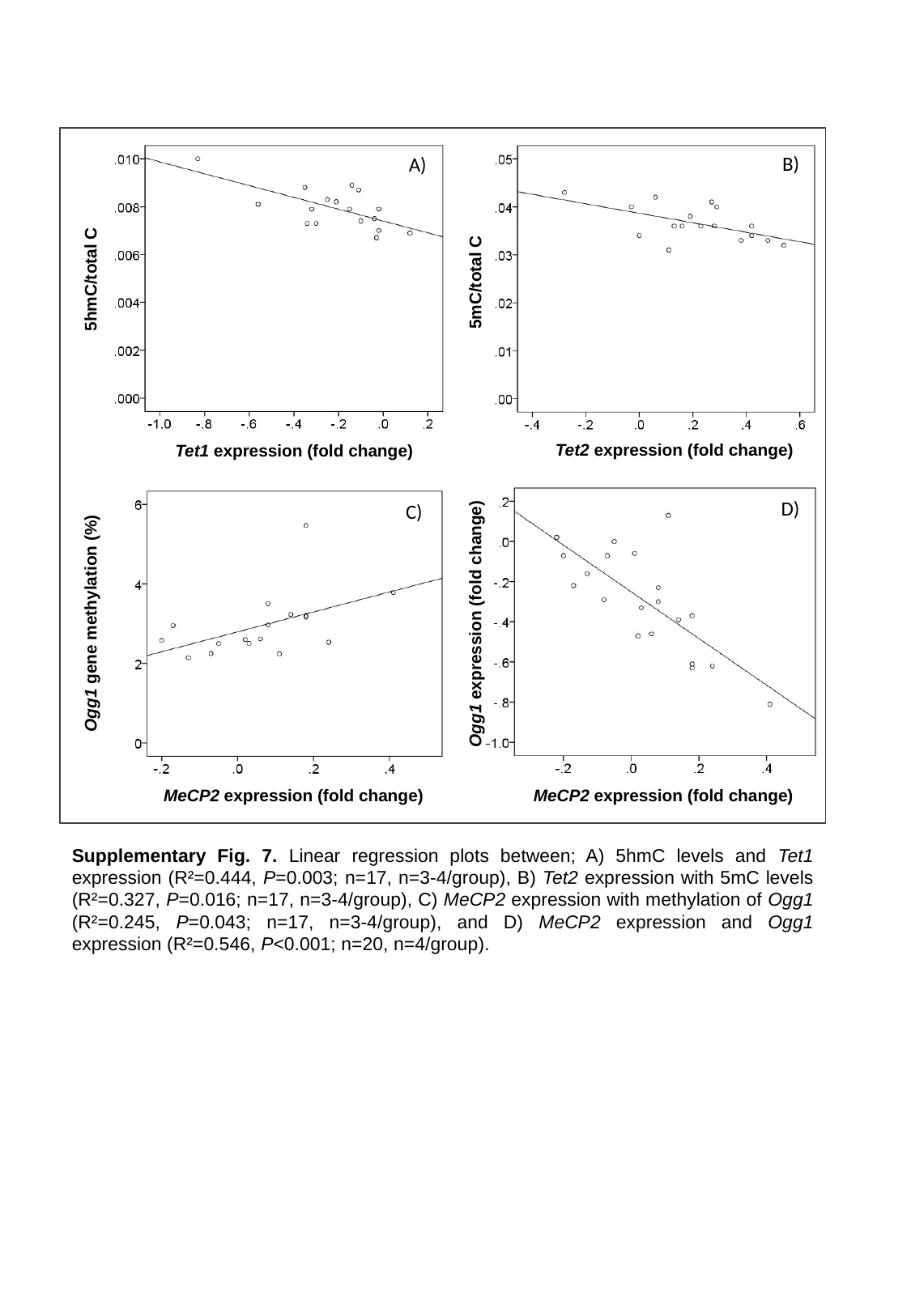

B)
A)
5hmC/total C
5mC/total C
Tet2 expression (fold change)
Tet1 expression (fold change)
D)
C)
Ogg1 gene methylation (%)
Ogg1 expression (fold change)
MeCP2 expression (fold change)
MeCP2 expression (fold change)
Supplementary Fig. 7. Linear regression plots between; A) 5hmC levels and Tet1 expression (R²=0.444, P=0.003; n=17, n=3-4/group), B) Tet2 expression with 5mC levels (R²=0.327, P=0.016; n=17, n=3-4/group), C) MeCP2 expression with methylation of Ogg1 (R²=0.245, P=0.043; n=17, n=3-4/group), and D) MeCP2 expression and Ogg1 expression (R²=0.546, P<0.001; n=20, n=4/group).
